# Predictors of post-stroke cognitive impairment using acute structural MRI neuroimaging: A systematic review and meta-analysis

**DOI:** 10.1177/17474930221120349

**Published:** 2022-09-12

**Authors:** Emily L Ball, Mahnoor Shah, Eilidh Ross, Rachel Sutherland, Charlotte Squires, Gillian E Mead, Joanna M Wardlaw, Terence J Quinn, Dorota Religa, Erik Lundström, Joshua Cheyne, Susan D Shenkin

**Affiliations:** 1 Centre for Clinical Brain Sciences, The University of Edinburgh, Edinburgh, UK; 2 Division of Clinical Geriatrics, Department of Neurobiology, Care Sciences and Society, Karolinska Institutet, Stockholm, Sweden; 3 NHS Lothian, Edinburgh, UK; 4 Ageing and Health Research Group, Usher Institute, The University of Edinburgh, Edinburgh, UK; 5 Centre for Clinical Brain Sciences, UK Dementia Research Institute, The University of Edinburgh, Edinburgh, UK; 6 Institute of Cardiovascular and Medical Sciences, University of Glasgow, Glasgow, UK; 7 Neurology, Department of Medical Sciences, Uppsala University, Uppsala, Sweden; 8 Advanced Care Research Centre, Usher Institute, The University of Edinburgh, Edinburgh, UK

**Keywords:** Stroke, cognitive impairment, dementia, neuroimaging, MRI

## Abstract

**Background:**

Stroke survivors are at an increased risk of developing post-stroke cognitive impairment and post-stroke dementia; those at risk could be identified by brain imaging routinely performed at stroke onset.

**Aim:**

This systematic review aimed to identify features which are associated with post-stroke cognitive impairment (including dementia) on magnetic resonance imaging (MRI) performed at stroke diagnosis.

**Summary of review:**

We searched the literature from inception to January 2022 and identified 10,284 records. We included studies that performed MRI at the time of stroke (0–30 days after a stroke) and assessed cognitive outcome at least 3 months after stroke. We synthesized findings from 26 papers, comprising 27 stroke-populations (N = 13,114, average age range = 40–80 years, 19–62% female). When data were available, we pooled unadjusted (OR_u_) and adjusted (OR_a_) odds ratios.

We found associations between cognitive outcomes and presence of cerebral atrophy (three studies, N = 453, OR_u_ = 2.48, 95% CI = 1.15–4.62), presence of microbleeds (two studies, N = 9151, OR_a_ = 1.36, 95% CI = 1.08–1.70), and increasing severity of white matter hyperintensities (three studies, N = 704, OR_a_ = 1.26, 95% CI = 1.06–1.49). Increasing cerebral small vessel disease score was associated with cognitive outcome following unadjusted analysis only (two studies, N = 499, OR_u_ = 1.34, 95%CI = 1.12–1.61; three studies, N = 950, OR_a_ = 1.23, 95% CI = 0.96–1.57). Associations remained after controlling for pre-stroke cognitive impairment. We did not find associations between other stroke features and cognitive outcome, or there were insufficient data.

**Conclusion:**

Acute stroke MRI features may enable healthcare professionals to identify patients at risk of post-stroke cognitive problems. However, there is still substantial uncertainty about the prognostic utility of acute MRI for this.

## Introduction

Cognitive problems after stroke are of major concern to stroke survivors and their families.^1^ Identifying who is at risk at the time of stroke may enable healthcare professionals to arrange appropriate follow-up, inform patients and their carers, and plan for possible future health outcomes. Individuals at risk of post-stroke cognitive problems could also be targeted for clinical trials with cognitive endpoints.

The cognitive consequences of stroke are conventionally described as post-stroke cognitive impairment (PSCI—impaired performance on a structured cognitive assessment) and the subcategory of post-stroke dementia (PSD—a clinical diagnosis of a cognitive change sufficient to interfere with daily life).

International guidelines for PSCI highlight that there are currently no prediction tools suitable for clinical practice.^2^ A survey of 60 UK healthcare professionals reported that respondents were aware that imaging features could predict PSCI, but they did not use these features in clinical practice.^3^ Acute stroke neuroimaging could help healthcare professionals to identify who is at risk of PSCI.

Acute stroke computed tomography (CT) brain imaging is routinely performed in clinical practice to determine the cause of stroke. CT brain imaging is inexpensive and quick to perform but has lower resolution than magnetic resonance imaging (MRI). Recently, MRI has become more available for stroke diagnosis in clinical practice. MRI also allows the identification of neuroimaging features such as cerebral microbleeds (CMB) that are rarely visible on CT brain scans. MRI may help identify neuroimaging features associated with post-stroke cognitive problems.

Cerebral small vessel disease (cSVD) is commonly associated with stroke and dementia.^4^ Neuroimaging features include white matter hyperintensities (WMH), CMB, lacunes, perivascular spaces (PVS), recent small subcortical infarcts, and cerebral atrophy.^5^ Three systematic reviews have described the associations between neuroimaging features and PSD/PSCI.^6–8^ One review found that stroke survivors with moderate to severe WMH had a two-to-three-fold increased risk in PSD/PSCI.^7^ Another review reported that medial temporal lobe atrophy (MTLA) and global atrophy were associated with increased risk of PSCI,^6^ and the third review highlighted an association between MTLA, WMH, and PSCI.^8^ These reviews included studies that performed brain imaging up to several months after a stroke, which does not reflect what happens in clinical practice. Only one review performed a sensitivity analysis comparing the association between severity of WMH and PSD when identified on CT versus MRI.^7^ The reviews did not report the association between acute stroke lesions and post-stroke cognitive outcome. However, a multicohort study of 2950 stroke survivors reported that infarcts in the left thalamus, left frontotemporal lobes, and right parietal lobe were associated with PSCI.^9^ Our previous systematic review focused on the prognostic utility of acute stroke CT finding that presence of atrophy, WMH, and pre-existing stroke lesions were associated with a two-to-three-fold increase in risk of PSD, and WMH was associated with a three-fold increased risk in PSCI.^10^ MRI is increasingly being used in clinical practice and is recommended for suspected TIA.^11^ A similar review focusing on MRI was needed.

### Aims

We determined whether features identifiable on brain MRI in acute stroke can predict PSD/PSCI. We included studies that performed MRI at the time of stroke. We extracted data from the published papers. As this review aimed to be directly applicable to clinical practice, we extracted neuroimaging features (acute stroke lesions and pre-existing stroke features) that could be visually rated on acute MR scans (e.g. presence/absence, severity scales, location).

## Methods

### Protocol and registration

We registered the protocol on PROSPERO (CRD42019128677). The review is reported according to PRISMA guidelines.^12^

### Eligibility criteria

Eligibility criteria are outlined in Table 1.

**Table 1.** Study inclusion criteria.

Study type:	Observational studies or clinical trials
Population:	• Intracerebral hemorrhage, ischemic and/or transient ischemic attack • Structural MR neuroimaging performed 0–30 days from index stroke
Prognostic factor:	• Neuroimaging features that are visually reported on MRI
Outcomes:	• Post-stroke cognitive impairment: assessed using a recognized cognitive tool • Post-stroke dementia: assessed using recognized diagnostic criteria • Cognitive outcome assessed at least 3 months after the stroke
Source:	• Published articles written in English quantifying the association between acute stroke neuroimaging features and cognitive outcome

MR: magnetic resonance; MRI: magnetic resonance imaging.

### Information sources

We designed a search strategy with an experienced librarian, combining terms relating to stroke, dementia/cognitive impairment, neuroimaging, and study type (Supplement 1). We searched electronic databases: Embase (OVID), MEDLINE (OVID), PsycINFO (EBSCO), and Cochrane Central Register of controlled Trials (CENTRAL) from inception to January 2022. We hand-searched the bibliographies of relevant reviews and included studies. We contacted study authors twice if it was not clear when brain imaging or cognitive follow-up were performed. If the authors did not respond, the study was excluded from the review.

### Study selection

We imported studies into Covidence software (Veritas Health Innovation Ltd).^13^ Two reviewers independently screened title/abstracts and then full text articles, and conflicts were resolved by consensus or by a third reviewer.

### Data collection process

We used a modified version of the CHARMS-PF checklist (CHecklist for critical Appraisal and data extraction for systematic Reviews of prediction Modeling Studies, tailored to Prognostic Factor studies);^14^ 12 (~50%) of the included articles were extracted by two reviewers. Disagreements were resolved by consensus or by another reviewer. As disagreements for 12 papers were minor, a single reviewer extracted data from the 14 remaining studies.

### Data extraction

We used a data extraction proforma (Supplement 2). If multiple papers included the same cohort, we used the study that presented data most relevant to our primary outcome. We extracted raw data, unadjusted and adjusted associations relating to neuroimaging features. Where various models were presented, we favored the model with the greatest number of variables.

### Neuroimaging features

We used the STandards for ReportIng Vascular changes on nEuroimaging (STRIVE) classification system to define neuroimaging features: atrophy, cSVD, WMH, lacunes, CMB, PVS, with additional categories of pre-existing stroke lesions (old infarcts or hemorrhages), acute stroke lesions (ischemic or hemorrhage, presence, number and location), and additional neuroimaging features (cortical superficial siderosis (cSS), hemorrhagic transformation, combinations of features).^5^

### Cognitive outcome

When studies performed cognitive assessments at multiple time points, we extracted data from the latest assessment after stroke. We produced harvest plots and performed meta-analysis only for studies that assessed global cognitive function/dementia.

### Harvest plot

These plots present associations between neuroimaging features and PSD/PSCI, after unadjusted or adjusted analysis, as well as the number of patients in each study and risk of bias.

### Meta-analysis

We included studies which reported data to allow calculation of unadjusted (OR_u_) or adjusted (OR_a_) associations in the meta-analysis. We log-transformed the OR and confidence intervals (CI) so that effect sizes were symmetrical around the null value and performed random-effects meta-analyses using the inverse-variance method. Variability due to between-study heterogeneity was quantified with I^2^. Due to heterogeneity between studies (measurement methods and reporting of data), a limited number of studies were suitable for meta-analysis. Where possible we dichotomized severity of neuroimaging features into presence/absence of these features.

We pooled studies that reported either PSD or PSCI, as there was considerable overlap between the definitions of these groups in different studies. We performed separate meta-analyses for studies that reported unadjusted or adjusted ORs.

We performed sensitivity analyses of studies that excluded patients with pre-stroke cognitive impairment/dementia (post hoc analysis), excluded hemorrhagic strokes (post hoc analysis), followed-up patients at least 6 months after stroke (planned analysis), and used a neuropsychological battery or diagnostic criteria (post hoc analysis). All analyses were performed using RStudio software (3.6.1).

### Quality assessment

We used the Quality in Prognostic factor Studies (QUIPS) tool to assess risk of bias.^14^

## Results

**Figure 1.**
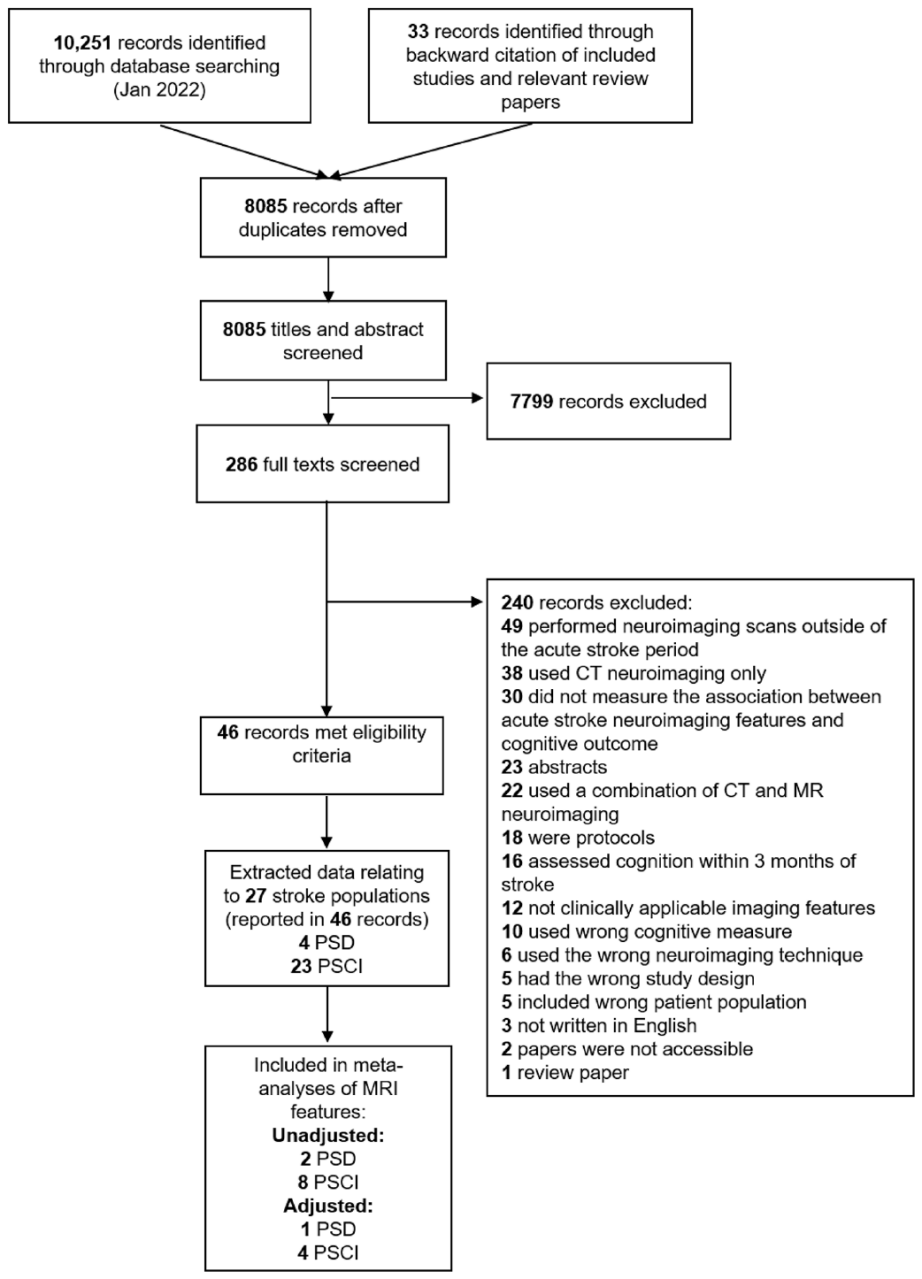
Study flow diagram.

We identified 10,284 records (Figure 1) and screened 286 full texts. Forty-six papers were eligible for inclusion (Supplement 3). Multiple papers reported the same stroke population. Findings from 26 papers, comprising 27 stroke-populations (N = 13,114, range of average ages = 40–80 years, 19–62% female) are synthesized in this review.^15–40^ Kandiah et al.^40^ contains two stroke cohorts, we refer to the development cohort as Kandiah et al.^41^ and the validation cohort as Kandiah et al.^42^

### Study characteristics

Studies included ischemic strokes (16 studies),^15,18,20–22,24–28,33,35,38,39,41,42^ hemorrhagic strokes (two studies),^31,36^ mixed strokes (three studies),^17,23,32^ and ischemic strokes and TIA (6 studies).^16,19,29,30,34,37^ Several of these studies only included patients with a particular stroke type or severity (Supplement 3). Twenty-one stroke-populations (78%) excluded patients with pre-stroke cognitive impairment and/or dementia.^16–18,20–22,24–29,31,33–39,41^ MRI was performed at various times from admission to 30 days. Full demographic and vascular risk factors for each stroke population are presented in Supplement 4.

### Cognitive assessment

Length of time from stroke to cognitive assessment ranged from 3 months to 7 years (Supplement 5). PSCI was the main cognitive outcome in 23 studies. Two of these studies reported impairment in specific cognitive domains only,^22,29^ the remainder assessed global cognitive function.^15–21,23,24,26,28,30,32–35,37–39,41,42^ Four studies reported diagnosis of dementia.^25,27,31,36^ Prevalence of PSCI/PSD ranged from 9% to 61%.

### Harvest plot

We summarized data on associations between neuroimaging features and PSCI or PSD from 23 stroke populations in the harvest plot,^15–21,23–26,28,30–32,34–39,41,42^ excluding two studies that only reported associations with specific cognitive domains^22,29^ and two studies which reported acute stroke features that did not align with our pre-specified classifications.^27,33^

### Atrophy

10 studies (N = 1475) measured global and/or localized atrophy (Supplement 6).^18,28,31,32,35,36,38,39,41,42^

**Figure 2.**
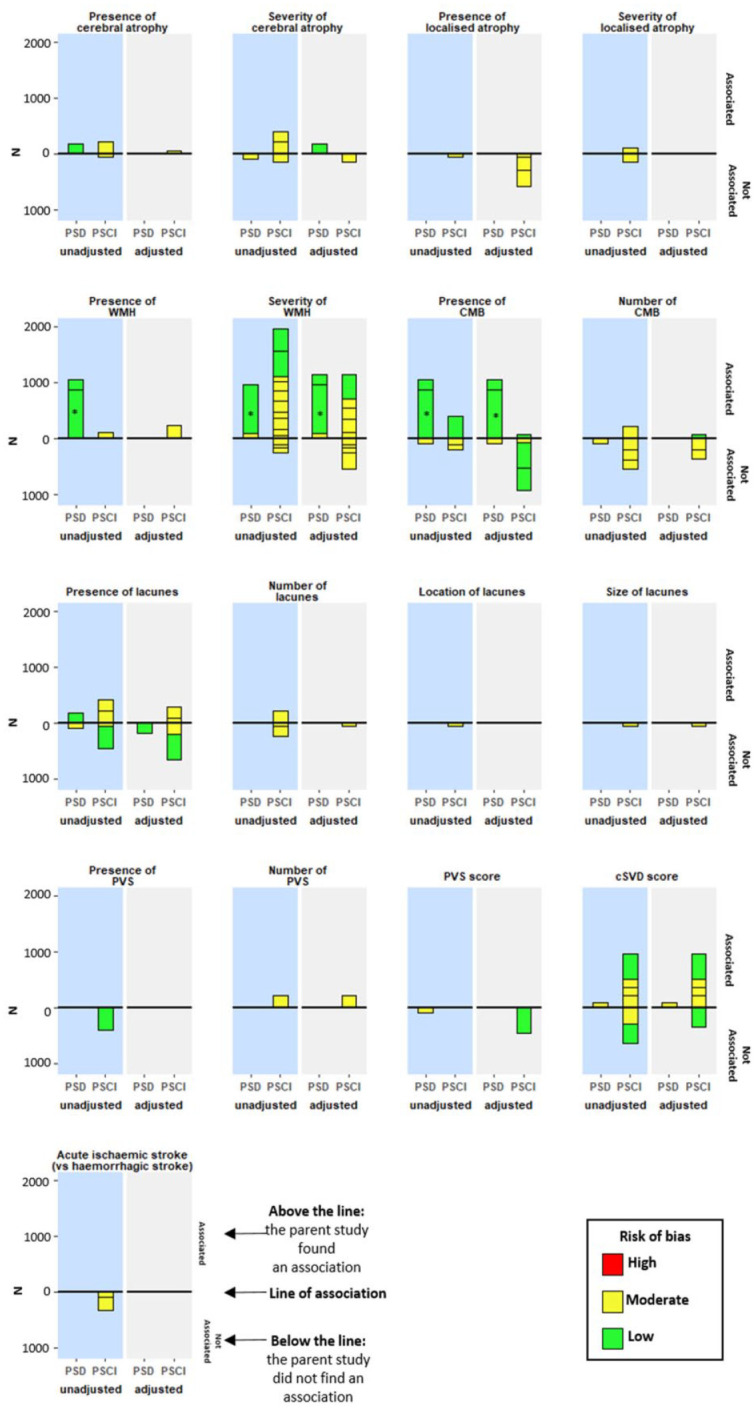
This harvest plot shows whether studies found an association between MRI features and cognitive outcome following unadjusted or adjusted analyses. PSD: post-stroke dementia; PSCI: post-stroke cognitive impairment; WMH: white matter hyperintensities; CMB: cerebral microbleeds; PVS: periventricular spaces; cSVD score: cerebral small vessel disease score.

The harvest plot (Figure 2) suggests an association between the presence of cerebral atrophy and PSCI/PSD, and our meta-analysis confirms this (3 studies, N = 453, OR_u_ = 2.48, 95% CI = 1.15–4.62, I^2^ = 0%, p = 0.004)^18,31,41^

As shown in the harvest plot, there was no clear association between severity of cerebral atrophy and PSCI/PSD (Figure 2) and data were too heterogeneous to meta-analyze.

There was no association between medial temporal lobe atrophy and PSCI (Figure 2).

### White matter hyperintensities

Twenty studies (N = 11,995) measured WMH (Supplement 7).^15–18,21–26,28,30–32,34,36,37,39,41,42^

Four studies found an association between the presence (versus absence) of WMH and PSCI/PSD (Figure 2). Data from three of these studies could be pooled, finding no association (3 studies, N = 8993, OR_u_ = 2.35, 95% CI = 0.92–6.01, I^2^ = 72%, p = 0.07).^25,31,34^

The harvest plot shows an association between WMH severity and PSCI/PSD (Figure 2). Three studies reported data suitable for meta-analysis and we found an association between WMH score and PSCI (three studies, N = 704, OR_a_ = 1.26, 95% CI = 1.06–1.49, I^2^ = 38%, p = 0.008; Figure 4).^17,26,28^ One study measured frontal executive impairment and found no association with WMH score (unadjusted).^22^

### Cerebral microbleeds

Fifteen studies (N = 11,060) measured CMB (Supplement 8).^19,21–23,25,26,28–31,34,36,39,41,42^

The harvest plot shows an association between the presence of CMB and PSD but not PSCI (Figure 2). Two studies were suitable for meta-analysis, finding association between the presence of CMB and PSCI/PSD (two studies, N = 9151, OR_a_ = 1.36, 95% CI = 1.08–1.70, I^2^ = 0%, p = 0.009).^25,26^ Two additional studies reported specific cognitive domains, one found an association with the presence of CMB, the other did not.^22,29^

There was no clear association between number of CMBs and PSCI/PSD (Figure 2).

### Lacunes

Nine studies (N = 1873) reported presence, number, location, and size of lacunes (Supplement 9 and Figure 2).^18,21,23,26,30,31,36,41,42^ Data from three studies were suitable for meta-analysis. There was no association between presence of lacunes and PSCI (three studies, N = 641, OR_u_ = 1.46, 95% CI = 0.96–2.23, I^2^ = 0%, p = 0.08).^18,30,31^

### Perivascular spaces

Four studies (N = 1153) reported PVS (Supplement 10),^23,26,30,36^ but there was insufficient evidence to form a conclusion (Figure 2).

### Cerebral small vessel disease

**Figure 3.**
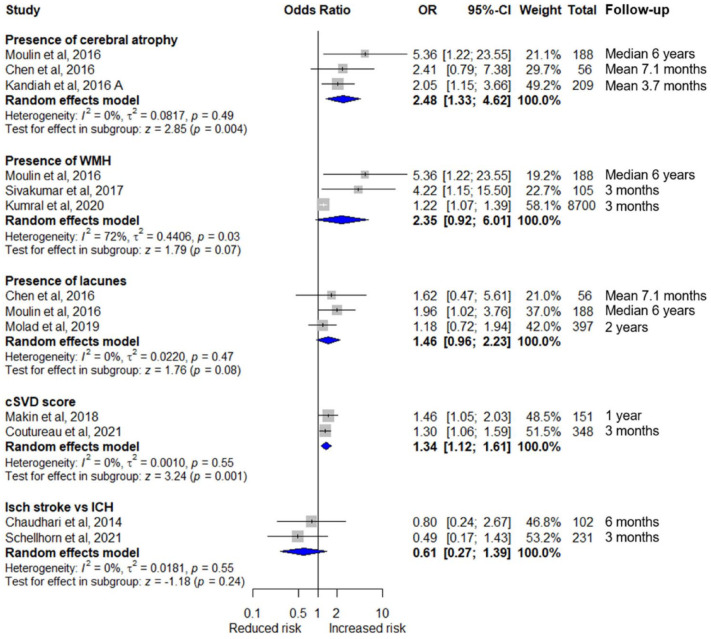
Unadjusted meta-analysis of neuroimaging features associated with cognitive outcome.

**Figure 4.**
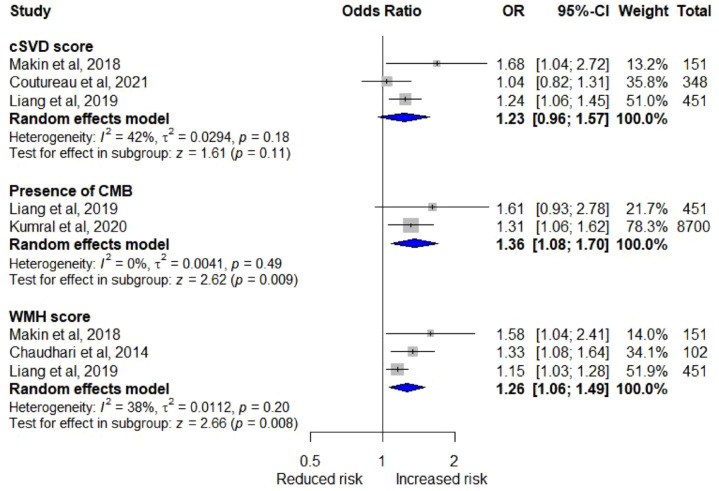
Adjusted meta-analysis of neuroimaging features associated with cognitive outcome.

Seven studies (N = 1510) reported cSVD score (Supplement 11).^20,23,26,28,35,36,38^ There was insufficient evidence reporting PSD. Four studies reported that increasing cSVD score is associated with PSCI (Figure 2). Meta-analysis found association for unadjusted data only (two studies, N = 499, OR_u_ = 1.34, 95% CI = 1.12–1.61, I^2^ = 0%, p = 0.001;^20,28^ Figure 3; three studies, N = 950, OR_a_ = 1.23, 95% CI = 0.96–1.57, I^2^ = 42%, p = 0.11; Figure 4).^20,26,28^

### Pre-existing stroke lesions

Five studies (N = 869) reported data relating to pre-existing stroke lesions (Supplement 12),^18,21,26,29,31^ there was no clear association with PSCI/PSD, although the neuroimaging features measured were heterogeneous (e.g. presence of old macrohemorrhage/lacunar infarct/cortical infarct).

### Acute stroke features

Two studies (N = 333) reported acute ischemic stroke (versus ICH) and found no association with PSCI (Figure 2);^17,32^ our meta-analysis confirms this (N = 333, OR_u_ = 0.61, 95% CI = 0.27–1.39, I^2^ = 0%, p = 0.24; Figure 3).

Seven studies (N = 9593) reported data relating to presence, number, and location of acute stroke lesions (Supplement 13).^17,25,27,32–34,36^ There was no clear association between acute stroke lesions and PSCI/PSD.

### Additional neuroimaging features

Four studies (four studies, N = 799) reported other neuroimaging features (cSS, hemorrhagic transformation, combinations of features (Supplement 14);^16,30,31,36^ due to the limited number of studies it was not possible to draw any conclusions about associations.

### Sensitivity analysis

After controlling for pre-stroke cognitive impairment, we also found a significant association between presence of lacunes and PSCI/PSD (two studies, N = 244, OR_u_ = 1.88, 95% CI = 1.06–3.35, I^2^ = 0%, p = 0.03)^18,31^. Results from the sensitivity analyses are presented in Supplement 15.

### Risk of bias

**Figure 5.**
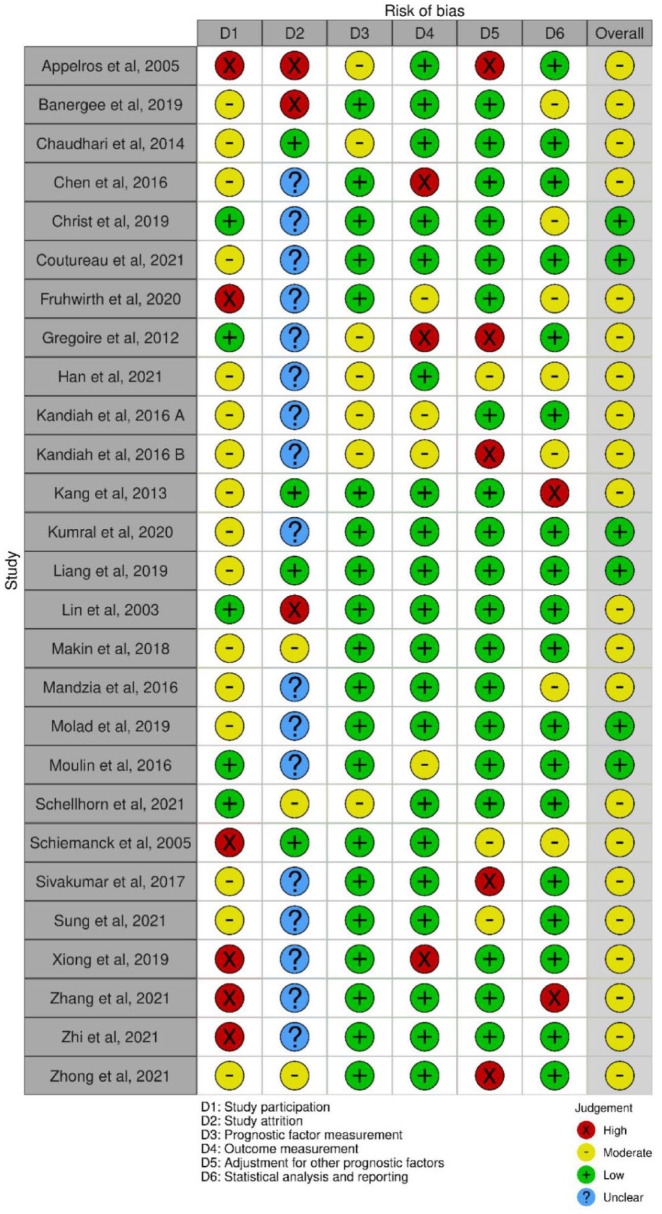
Risk of bias plot.

We rated no studies with high overall risk of bias (Figure 5). Issues with external validity were common due to studies including only specific stroke types (e.g. lacunar stroke, middle cerebral artery lesion only) and excluding more severe strokes. The majority of studies did not clearly report the reasons for loss to follow-up.

## Discussion

### Key findings

This systematic review included 27 cohorts of patients with stroke (N = 13,114). Features of cSVD, visible on acute stroke MRI, were associated with PSCI/PSD. The presence of cerebral atrophy, presence and severity of WMH, presence of CMB, and total cSVD score were associated with increased risk of either PSCI and/or PSD. More severe WMH (adjusted), worse cSVD (unadjusted), presence of cerebral atrophy (unadjusted), and presence of CMB (adjusted) were associated with PSCI/PSD in meta-analyses. We did not find associations between other features and PSCI/PSD or there was insufficient evidence to draw a conclusion. Heterogeneity between studies limited the potential to pool data.

We aimed to explore whether routine MRI collected for clinical purposes at the time of stroke also have a use in predicting long-term cognitive impairment. This is the first systematic review to address the question of whether MRI taken at the time of stroke is useful for identifying patients at risk of post-stroke cognitive problems. Previous systematic reviews included studies that performed brain scans up to several months after stroke. In agreement with these reviews, we also found that WMH were associated with poorer cognitive outcome.^7,8^ Crucially, our review looked at pre-existing features and acute stroke lesions visually reported at the time of stroke—finding that pre-existing features were more clearly related than acute lesions to cognitive outcomes—and has clinical implications for early identification of patients at increased risk of PSCI.

### Strengths and limitations of this systematic review

In order for our findings to be clinically applicable, we only included neuroimaging features that could be assessed by clinicians, and not those using computerized methods which would require specialist facilities, analysis, and extra time. Although we included brain scans performed within 30 days after a stroke, 78% of the included studies performed scans during acute stroke or within 1 week of the stroke. Studies that assessed PSCI often did not attempt to diagnose dementia, meaning that “PSCI” could include people with mild cognitive impairment or those with dementia. We combined studies that assessed either PSCI or PSD in the same meta-analysis. We did, however, include studies which measured PSCI or PSD separately in our harvest plot, showing association with presence of WMH and CMB and PSD. Dementia was the main cognitive outcome of only four of the included studies; therefore, we can draw limited conclusions from these data. Our review was limited to studies written in English, but we did not restrict the search by language; therefore, we are aware that we were unable to include three studies written in Chinese or Japanese.

### Strengths and limitations of included studies

Many of the included studies defined neuroimaging features according to STRIVE criteria which helped when synthesizing findings.^5^ However, studies used different measurement methods (presence/severity/location) and analysis techniques (unadjusted/adjusted) to assess the association with cognitive outcome (PSD/PSCI/specific cognitive domains).

Most studies were small in size. Several studies also excluded patients who could not provide informed consent, or who had aphasia/communication difficulties; therefore, findings may not be applicable to patients with more severe strokes.

### Research implications

To aid with synthesizing neuroimaging features, studies should provide definitions of the neuroimaging features they are measuring (e.g. STRIVE criteria) and use validated scales. Published guidance on reporting location of acute stroke lesions would be advantageous but do not currently exist. To distinguish which neuroimaging features are associated with PSCI (no dementia) compared to PSD, studies could diagnose according to *the Diagnostic and Statistical Manual of Mental Disorders* (5th ed.; *DSM*-5) criteria for major and minor neurocognitive disorder, although reporting full results of cognitive and functional tests is also useful.

### Clinical implications

In conjunction with other clinical risk factors such as low education, atrial fibrillation, hypercholesterolemia, and prior stroke (Supplement 16), having a structured way of reporting acute stroke brain scans in clinical practice, that is quick to perform, may help healthcare professionals to identify who is at risk of post-stroke cognitive problems. Should it become possible to identify which stroke survivors are at risk of cognitive problems, future studies need to explore how best to communicate this information to patients and their families.

### Conclusions

Routinely performed acute stroke MRI may help healthcare professionals to identify which stroke survivors have an increased risk of post-stroke cognitive problems, but overall effect size is small. Understanding whether patients with acute stroke would want to know this prognostic information, and how best to support them, requires further research.

## Supplemental Material

sj-docx-1-wso-10.1177_17474930221120349 – Supplemental material for Predictors of post-stroke cognitive impairment using acute structural MRI neuroimaging: A systematic review and meta-analysis Supplemental material, sj-docx-1-wso-10.1177_17474930221120349 for Predictors of post-stroke cognitive impairment using acute structural MRI neuroimaging: A systematic review and meta-analysis by Emily L Ball, Mahnoor Shah, Eilidh Ross, Rachel Sutherland, Charlotte Squires, Gillian E Mead, Joanna M Wardlaw, Terence J Quinn, Dorota Religa, Erik Lundström, Joshua Cheyne and Susan D Shenkin in International Journal of Stroke
