# Supplementary material for: Predictors of post-stroke cognitive impairment using acute structural MRI neuroimaging: A systematic review and meta-analysis
Source: Int J Stroke. 2022 Sep 12;18(5):543–54. doi: 10.1177/17474930221120349 (PMC10201083; doi:10.1177/17474930221120349)
Supplement: sj-docx-1-wso-10.1177_17474930221120349 – Supplemental material for Predictors of post-stroke cognitive impairment using acute structural MRI neuroimaging: A systematic review and meta-analysis [file sj-docx-1-wso-10.1177_17474930221120349.docx]

**Supplementary Online Content**

**[Supplement 1: Search strategy](#_Toc112944637)** [2](#_Toc112944637)

[**Supplement 2: Data extraction form** 8](#_Toc112944638)

[**Supplement 3: Study characteristics of included studies** 11](#_Toc112944639)

[**Supplement 4: Clinical risk factors of stroke population included in cognitive follow-up** 15](#_Toc112944640)

[**Supplement 5: Cognitive assessment methods** 20](#_Toc112944641)

[**Supplement 6: Atrophy** 22](#_Toc112944642)

[**Supplement 7: White matter hyperintensities** 29](#_Toc112944643)

[**Supplement 8: Cerebral microbleeds** 37](#_Toc112944644)

[**Supplement 9: Lacunes** 45](#_Toc112944645)

[**Supplement 10: Perivascular spaces** 54](#_Toc112944646)

[**Supplement 11: Cerebral small vessel disease** 57](#_Toc112944647)

[**Supplement 12: Pre-existing stroke lesions** 62](#_Toc112944648)

[**Supplement 13: Acute stroke features** 66](#_Toc112944649)

[**Supplement 14: Additional neuroimaging features** 75](#_Toc112944650)

[**Supplement 15: Sensitivity Analysis** 79](#_Toc112944651)

[**Supplement 16: Meta-analysis of risk factors associated with PSCI/PSD (unadjusted prognostic factors)** 81](#_Toc112944652)

This supplementary material has been provided by the authors to give readers additional information about their work.

# **Supplement 1: Search strategy**

**Medline**

**Stroke**

1. cerebrovascular disorders/ or exp basal ganglia cerebrovascular disease/ or brain ischemia/ or hypoxia-ischemia, brain/ or ischemic attack, transient/ or carotid artery diseases/ or carotid artery thrombosis/ or intracranial arterial diseases/ or cerebral arterial diseases/ or exp "intracranial embolism and thrombosis"/ or exp intracranial hemorrhages/ or exp stroke/
2. (stroke$ or apoplex$ or cerebral vasc$ or cerebrovasc$ or cva or transient isch$mic attack$ or tia).tw.
3. ((brain or cerebr$ or cerebell$ or hemispher$ or intracran$ or intracerebral or infratentorial or supratentorial or space-occupying) adj5 (isch?emi$ or infarct$ or thrombo$ or emboli$ or occlus$ or hypoxi$)).tw.
4. ((brain or cerebr$ or cerebell$ or hemispher$ or intracran$ or intracerebral or infratentorial or supratentorial or intraventricular) adj5 (h?emorrhag$ or h?ematoma$ or bleed$)).tw.
5. or/1-4

**Dementia and cognitive impairment**

1. exp dementia/ or memory disorders/ or neurocognitive disorders/ or cognition disorders/ or cognitive dysfunction/
2. dement$.tw.
3. ((attention or awareness or cognit$ or neuropsych$ or neurocognit$ or neurobehav$ or psycholog$ or memor$ or recall or think$) adj5 (declin$ or impair$ or domain$ or test$ or assess$ or function$ or batter$ or disorder$ or dysfunct$ or deficit$ or declin$ or abilit$ or problem$ or difficult$ or disturbance$ or disabilit$)).tw.
4. mental processes/ or cognition/ or awareness/ or cognitive reserve/ or executive function/ or learning/ or thinking/ or perception/ or memory/
5. exp psychological tests/
6. MMSE.tw.
7. or/6-11

**Neuroimaging**

1. diagnostic imaging/ or neuroimaging/ or multimodal imaging/
2. tomography, x-ray computed/
3. ((compute$ or cerebral or CAT or CT or brain) adj5 (imag$ or scan$ or neuroimag$ or tomogra$ or marker$ or feature$ or x-ray)).tw.
4. Magnetic resonance imaging/
5. (MRI or MRi or NMRI or NMRi).tw.
6. ((magn$ or resonance or MR or MTC or MT or NMR) adj5 (imag$ or scan$ or neuroimag$ or tomogra$ or marker$ or feature$)).tw.
7. or/13-18

**Study type**

1. prognosis/
2. observational study/
3. randomized controlled trial.pt.
4. controlled clinical trial.pt.
5. Epidemiologic Studies/
6. exp Case-Control Studies/
7. exp Cohort Studies/
8. (epidemiologic adj (study or studies)).ab,ti.
9. case control.ab,ti.
10. (cohort adj (study or studies)).ab,ti.
11. cohort analy$.ab,ti.
12. (follow up adj (study or studies)).ab,ti.
13. longitudinal.ab,ti.
14. retrospective$.ab,ti.
15. prospective$.ab,ti.
16. (observ$ adj3 (study or studies)).ab,ti.
17. or/20-35

**Running the search**

1. 5 and 12 and 19 and 36

**EMBASE**

**Stroke**

1. cerebrovascular disease/ or exp basal ganglion hemorrhage/ or brain ischemia/ or hypoxic ischemic encephalopathy/ or transient ischemic attack/ or carotid artery disease/ or carotid artery thrombosis/ or cerebral artery disease/ or brain embolism/ or occlusive cerebrovascular disease/ or cerebral sinus thrombosis/ or exp brain hemorrhage/ or hypophysis apoplexy/ or exp brain hematoma/ or epidural hematoma/ or exp cerebrovascular accident/ or exp brain infarction/
2. (stroke$ or apoplex$ or cerebral vasc$ or cerebrovasc$ or cva or transient isch$mic attack$ or tia).tw.
3. ((brain or cerebr$ or cerebell$ or hemispher$ or intracran$ or intracerebral or infratentorial or supratentorial or space-occupying) adj5 (isch?emi$ or infarct$ or thrombo$ or emboli$ or occlus$ or hypoxi$)).tw.
4. ((brain or cerebr$ or cerebell$ or hemispher$ or intracran$ or intracerebral or infratentorial or supratentorial or intraventricular) adj5 (h?emorrhag$ or h?ematoma$ or bleed$)).tw.
5. or/1-4

**Dementia and cognitive impairment**

1. exp dementia/ or memory disorder/ or disorders of higher cerebral function/ or cognitive defect/
2. dement$.tw.
3. ((attention or awareness or cognit$ or neuropsych$ or neurocognit$ or neurobehav$ or psycholog$ or memor$ or recall or think$) adj5 (declin$ or impair$ or domain$ or test$ or assess$ or function$ or batter$ or disorder$ or dysfunct$ or deficit$ or declin$ or abilit$ or problem$ or difficult$ or disturbance$ or disabilit$)).tw.
4. mental function/ or cognition/ or awareness/ or cognitive reserve/ or executive function/ or learning/ or thinking/ or perception/ or memory/
5. exp psychologic test/ or exp dementia assessment/ or exp cognition assessment/
6. MMSE.tw
7. or/6-11

**Neuroimaging**

1. diagnostic imaging/ or neuroimaging/ or multimodal imaging/
2. x-ray computed tomography/ or computer assisted tomography/ or high resolution computer tomography/
3. ((compute$ or cerebral or CAT or CT or brain) adj5 (imag$ or scan$ or neuroimag$ or tomogra$ or marker$ or feature$ or x-ray)).tw.
4. nuclear magnetic resonance imaging/
5. (MRI or MRi or NMRI or NMRi).tw.
6. ((magn$ or resonance or MR or MTC or MT or NMR) adj5 (imag$ or scan$ or neuroimag$ or tomogra$ or marker$ or feature$)).tw.
7. or/13-18

**Study type**

1. prognosis/
2. observational study/
3. randomized controlled trial.pt.
4. controlled clinical trial.pt.
5. exp case control study/
6. cohort analysis/
7. (epidemiologic adj (study or studies)).ab,ti.
8. case control.ab,ti.
9. (cohort adj (study or studies)).ab,ti.
10. cohort analy$.ab,ti.
11. (follow up adj (study or studies)).ab,ti.
12. longitudinal.ab,ti.
13. retrospective$.ab,ti.
14. prospective$.ab,ti.
15. (observ$ adj3 (study or studies)).ab,ti.
16. or/20-34

**Running the search**

1. 5 and 12 and 19 and 35

**PsycINFO**

**Stroke**

1. cerebrovascular disorders/ or cerebral ischemia/ or cerebral hemorrhage/ or cerebrovascular accidents/
2. (basal gangli$ h?morrhage$ or carotid artery disease$ or carotid artery thrombo$ or intracranial arter$ disease$ or cerebral arter$ disease$).tw.
3. (stroke$ or apoplex$ or cerebral vasc$ or cerebrovasc$ or cva or transient isch$mic attack$ or tia).tw.
4. ((brain or cerebr$ or cerebell$ or hemispher$ or intracran$ or intracerebral or infratentorial or supratentorial or space-occupying) adj5 (isch?emi$ or infarct$ or thrombo$ or emboli$ or occlus$ or hypoxi$)).tw.
5. ((brain or cerebr$ or cerebell$ or hemispher$ or intracran$ or intracerebral or infratentorial or supratentorial or intraventricular) adj5 (h?emorrhag$ or h?ematoma$ or bleed$)).tw.
6. or/1-5

**Dementia and cognitive impairment**

1. exp dementia/ or memory disorders/ or cognitive impairment/
2. dement$.tw.
3. ((attention or awareness or cognit$ or neuropsych$ or neurocognit$ or neurobehav$ or psycholog$ or memor$ or recall or think$) adj5 (declin$ or impair$ or domain$ or test$ or assess$ or function$ or batter$ or disorder$ or dysfunct$ or deficit$ or declin$ or abilit$ or problem$ or difficult$ or disturbance$ or disabilit$)).tw.
4. cognitive processes/ or cognition/ or awareness/ or cognitive reserve/ or executive function/ or learning/ or thinking/ or perception/ or memory/
5. exp psychological assessment/
6. MMSE.tw.
7. or/7-12

**Neuroimaging**

1. neuroimaging/
2. multi$modal imag$.tw.
3. ((compute$ or cerebral or CAT or CT or brain) adj5 (imag$ or scan$ or neuroimag$ or tomogra$ or marker$ or feature$ or x-ray)).tw.
4. magnetic resonance imaging/
5. (MRI or MRi or NMRI or NMRi).tw.
6. ((magn$ or resonance or MR or MTC or MT or NMR) adj5 (imag$ or scan$ or neuroimag$ or tomogra$ or marker$ or feature$)).tw.
7. or/14-19

**Study type**

1. prognosis/
2. observation methods/
3. randomized controlled trial.pt.
4. controlled clinical trial.pt.
5. exp cohort analysis/
6. (epidemiologic adj (study or studies)).ab,ti.
7. case control.ab,ti.
8. (cohort adj (study or studies)).ab,ti.
9. cohort analy$.ab,ti.
10. (follow up adj (study or studies)).ab,ti.
11. longitudinal.ab,ti.
12. retrospective$.ab,ti.
13. prospective$.ab,ti.
14. (observ$ adj3 (study or studies)).ab,ti.
15. or/21-34

**Running the search**

1. 6 and 13 and 20 and 35

**Cochrane Central Register of controlled Trials (CENTRAL)**

**Stroke**

1. MeSH descriptor: [Cerebrovascular Disorders] this term only
2. MeSH descriptor: [Basal Ganglia Cerebrovascular Disease] explode all trees
3. MeSH descriptor: [Brain Ischemia] this term only
4. MeSH descriptor: [Hypoxia-Ischemia, Brain] this term only
5. MeSH descriptor: [Ischemic Attack, Transient] this term only
6. MeSH descriptor: [Carotid Artery Diseases] this term only
7. MeSH descriptor: [Carotid Artery Thrombosis] this term only
8. MeSH descriptor: [Intracranial Arterial Diseases] this term only
9. MeSH descriptor: [Cerebral Arterial Diseases] this term only
10. MeSH descriptor: [Intracranial Embolism and Thrombosis] explode all trees
11. MeSH descriptor: [Intracranial Hemorrhages] explode all trees
12. MeSH descriptor: [Stroke] explode all trees
13. ((stroke* or apoplex* or cerebral vasc* or cerebrovasc* or cva or transient isch*mic attack* or tia))
14. ((brain or cerebr* or cerebell* or hemispher* or intracran* or intracerebral or infratentorial or supratentorial or space-occupying) NEAR/3 (isch*emi* or infarct* or thrombo* or emboli* or occlus* or hypoxi*))
15. (((brain or cerebr* or cerebell* or hemispher* or intracran* or intracerebral or infratentorial or supratentorial or intraventricular) NEAR/3 (h*morrhag* or h*matoma* or bleed*)))
16. #1 OR #2 OR #3 OR #4 OR #5 OR #6 OR #7 OR #8 OR #9 OR #10 OR #11 OR #12 OR #13 OR #14 OR #15

**Dementia and cognitive impairment**

1. MeSH descriptor: [Dementia] explode all trees
2. MeSH descriptor: [Memory Disorders] this term only
3. MeSH descriptor: [Neurocognitive Disorders] this term only
4. MeSH descriptor: [Cognition Disorders] this term only
5. MeSH descriptor: [Cognitive Dysfunction] this term only
6. (dement*)
7. (((attention or awareness or cognit* or neuropsych* or neurocognit* or neurobehav* or psycholog* or memor* or recall or think*) NEAR/3 (declin* or impair* or domain* or test* or assess* or function* or batter* or disorder* or dysfunct* or deficit* or declin* or abilit* or problem* or difficult* or disturbance* or disabilit*)))
8. MeSH descriptor: [Mental Processes] this term only
9. MeSH descriptor: [Cognition] this term only
10. MeSH descriptor: [Awareness] this term only
11. MeSH descriptor: [Cognitive Reserve] this term only
12. MeSH descriptor: [Executive Function] this term only
13. MeSH descriptor: [Learning] explode all trees
14. MeSH descriptor: [Thinking] this term only
15. MeSH descriptor: [Perception] this term only
16. MeSH descriptor: [Memory] this term only
17. MeSH descriptor: [Psychological Tests] explode all trees
18. (MMSE)
19. #17 OR #18 OR #19 OR #20 OR #21 OR #22 OR #23 OR #24 OR #25 OR #26 OR #27 OR #28 OR #29 OR #30 OR #31 OR #32 OR #33 OR #34

**Neuroimaging**

1. MeSH descriptor: [Diagnostic Imaging] this term only
2. MeSH descriptor: [Neuroimaging] this term only
3. MeSH descriptor: [Multimodal Imaging] this term only
4. MeSH descriptor: [Tomography, X-Ray Computed] this term only
5. (((compute* or cerebral or CAT or CT or brain) NEAR/3 (imag* or scan* or neuroimag* or tomogra* or marker* or feature* or x-ray)))
6. MeSH descriptor: [Magnetic Resonance Imaging] this term only
7. ((MRI or MRi or NMRI or NMRi))
8. (((magn* or resonance or MR or MTC or MT or NMR) NEAR/3 (imag* or scan* or neuroimag* or tomogra* or marker* or feature*)))
9. #36 OR #37 OR #38 OR #39 OR #40 OR #41 OR #42 OR #43

**Running the search**

1. #16 AND #35 AND #44

# **Supplement 2: Data extraction form**

**Study characteristics:**

| **Year of stroke** | **Country** | **Setting**  **(e.g. inpatient, outpatient)** | **Followed-up/recruited (%)** | **Females**  **(%)** | **Mean age Years (SD)** | **Stroke type**  **(IS/ICH/TIA/Lacunar)** | **Excluded**  **pre-stroke cognitive impairment**  **or**  **dementia** | **Excluded prior stroke**  **(Yes/No)** | **How long after stroke was the scan performed?** | **Neuroimaging features reported** | **Who rated the**  **MRI scans?** | **MRI scanner strength** |
| --- | --- | --- | --- | --- | --- | --- | --- | --- | --- | --- | --- | --- |
|  |  |  |  |  |  |  |  |  |  |  |  |  |

**Cognitive follow-up:**

| **Latest follow-up time** | **PSD**  **N (%)** | **PSCI**  **N (%)** | **Diagnostic criteria for PSD** | **Criteria for PSCI** |
| --- | --- | --- | --- | --- |
|  |  |  |  |  |

**Clinical and vascular risk factors of patients included in the latest follow-up:**

| **Age** | **Females**  **N (%)** | **Education** | **Hypertension**  **(%)** | **Diabetes (%)** | **Hypercholesterolemia (%)** | **Atrial fibrillation (%)** | **Smoking (%)** | **Alcohol intake (%)** | **APOE status (%)** | **Ischaemic heart disease**  **(%)** | **Prior stroke/TIA (%)** | **Stroke severity** | **Proportion with cognitive impairment prior to stroke (%)** |
| --- | --- | --- | --- | --- | --- | --- | --- | --- | --- | --- | --- | --- | --- |
|  |  |  |  |  |  |  |  |  |  |  |  |  |  |

**Neuroimaging features:**

|  | | | | | **Unadjusted analysis** | | | | **Adjusted analysis** | | | |
| --- | --- | --- | --- | --- | --- | --- | --- | --- | --- | --- | --- | --- |
| **Neuroimaging feature** | **MRI Sequence** | **Measurement technique** | **Developed**  **PSD/PSCI** | **Not developed PSD/PSCI** | **Effect size** | **Sig. level** | **Statistical test** | **Associated with PSD/PSCI** | **Effect size** | **Sig. level** | **Statistical test** | **Associated with PSD/PSCI** |
|  |  |  |  |  |  |  |  |  |  |  |  |  |

**Data for meta-analyses:**

| **Risk factor** | **Definition** | **Biomarker present & developed PSD/PSCI** | **Total that developed PSD/PSCI** | **Biomarker present & did not develop PSD/PSCI** | **Total that did not develop PSD/PSCI** | **OR presented in study** |
| --- | --- | --- | --- | --- | --- | --- |
| Female |  |  |  |  |  |  |
| Low education |  |  |  |  |  |  |
| Hypertension |  |  |  |  |  |  |
| Diabetes |  |  |  |  |  |  |
| Hypercholesterolemia |  |  |  |  |  |  |
| Atrial fibrillation |  |  |  |  |  |  |
| Smoking |  |  |  |  |  |  |
| High alcohol intake |  |  |  |  |  |  |
| APOE |  |  |  |  |  |  |
| Ischaemic heart disease |  |  |  |  |  |  |
| Prior stroke |  |  |  |  |  |  |
| Prior TIA |  |  |  |  |  |  |
| Acute ischaemic stroke  (vs. ICH) |  |  |  |  |  |  |
| Presence of cerebral atrophy |  |  |  |  |  |  |
| Presence of lacunes |  |  |  |  |  |  |
| Presence of WMH |  |  |  |  |  |  |
| cSVD score |  |  |  |  |  |  |

**Risk of bias assessment:**

| **Author** | **Study participation**  **Low risk:** Broad range of stroke types (few exclusion criteria)  **Moderate risk:** Ischaemic and/or ICH (excluded key risk factors)  **High risk:** Specific type of stroke (e.g. lacunar only) | **Study attrition**  **Low risk:** No difference between participants who dropped out and those who were included in follow-up  **Moderate risk:** There was a difference in a few risk factors between those who dropped out and were included (e.g. age only)  **High risk:** There were several differences in risk factors between those who dropped out and were included | **Prognostic factor measurement**  **Low risk:** CT scan performed at the same time point after stroke  **Moderate risk:** Some variability about when the scan was performed (e.g. within a month)  **High risk**: Large variability about when the scan was performed | **Outcome measurement**  **Low risk**: Cognition was assessed using the same tests/criteria, performed in the same setting (e.g. face to face) for each participant  **Moderate risk:** Cognition was assessed using different tests/criteria in the same or different settings (e.g. some by phone) for each participant  **High risk:**  Cognition was assessed using different tests/criteria in different settings (e.g. some by phone) and asked relatives rather than interviewing the participant | **Adjustment for other prognostic factors**  **Low risk:** Imaging findings were adjusted for key risk factors (e.g. age, sex or risk factors identified in a statistical model)  **Moderate risk:** Imaging findings were adjusted for some key risk factors (e.g. age only)  **High risk:** Imaging findings were not adjusted for any key risk factors | **Statistical analysis and reporting**  **Low risk**: N and/or effect size reported, p-value and statistical test reported. No missing data.  **Moderate risk:** N and/or effect size not reported, or only p-values reported, statistical test reported  **High risk**: only p-values reported, no statistical test reported, no statistics reported | **Overall**  **risk of bias** |
| --- | --- | --- | --- | --- | --- | --- | --- |
|  |  |  |  |  |  |  |  |

# **Supplement 3: Study characteristics of included studies**

27 stroke-populations^1-26^ (described in 46 papers)^1-46^ were eligible for inclusion.

| **Study, year** | **Year of stroke** | **Country** | **Setting** | **Followed-up / recruited or screened (%)** | **Females (%)** | **Mean age**  **Years (SD)** | **Stroke type** | **Excluded pre-stroke cognitive impairment or dementia** | **Excluded prior stroke** | **Neuroimaging features reported** | **MRI scanner strength** | **Performed adjusted analyses** |
| --- | --- | --- | --- | --- | --- | --- | --- | --- | --- | --- | --- | --- |
| Appelros et al, 2005 | 1989-1992 | Sweden | Inpatient^[[1]](#footnote-1)^ | 56/81  (69%) | NS | NS | IS^[[2]](#footnote-2)^ | No | Yes | WMH | 1.0T^[[3]](#footnote-3)^ | No |
| Banergee et al, 2019 | NS | UK/Netherlands | Inpatient1 | 117/383  (31%) | 45/117 (38%) | 73.1 (9.1) | IS & TIA^[[4]](#footnote-4)^ | Yes20 5 | No | WMH, additional features | 1.5T/3T | Yes |
| Chaudhari et al, 2014 | 2011-2013 | India | Inpatient | 102/106  (96%) | 27/102 (26%) | 59.4 (10.9) | IS &  ICH | Yes20 | No | Acute stroke lesions, WMH | NS | Yes |
| Chen et al, 2016 | 2013 | China | Inpatient | 56/165  (34%) | 21/56 (38%) | 63.8 (8.3) | IS | Yes^[[5]](#footnote-5)^ | Yes^[[6]](#footnote-6)^ | Atrophy, lacunes, pre-existing stroke lesions, WMH | 1.5T/3.0T | Yes |
| Christ et al, 2019 | NS | Germany | Inpatient | 66/NS (NS%) | 30/66 (45%) | NS | IS & TIA | No | No | CMB | NS | Yes |
| Coutureau et al, 2021^[[7]](#footnote-7)^ | 2012-2015 | France | Inpatient1 | 348/428  (81%) | 127/348 (36%) | 67.5 (14.1) | IS^[[8]](#footnote-8)^ | Yes^[[9]](#footnote-9)^ | Yes | cSVD | 3T | Yes |
| Fruhwirth et al, 2020 | 2012-2017 | Austria | Inpatient | 82/115  (71%) | 19/82 (23%) | 61 (10) | IS^[[10]](#footnote-10)^ | Yes5 | No | CMB, lacunes, pre-existing stroke lesions, WMH | 1.5T | Yes |
| Gregoire et al, 2012 | 2001-2002 | England | Outpatient | 26/55  (47%) | 10/26 (38%) | NS | IS | Yes^[[11]](#footnote-11)^ | No | CMB, WMH | 1.5T | No |
| Han et al, 2021 | 2017-2020 | China | Inpatient | 208/NS  (NS%) | 79/208  (38%) | Range: 40-80 years | IS & ICH^[[12]](#footnote-12)^ | No^[[13]](#footnote-13)^ | No | CMB, cSVD, lacunes, PVS, WMH | 3T | Yes |
| Kandiah et al, 2016 A | 2008-2012 | Singapore | Outpatient | 209/243  (86%) | 67/209  (32%) | 61.67  (12.46) | IS14 | Yes5 | No | Atrophy, CMB, lacunes, WMH | NS | Yes |
| Kandiah et al, 2016 B | 2012-2014 | Singapore | Outpatient | 89/185  (48%) | NS | NS | IS^[[14]](#footnote-14)^ | NS | No | Atrophy, CMB, lacunes, WMH | NS | No |
| Kang et al, 2013 | 2006-2009 | South Korea | Inpatient | 240/423  (57%) | NS | NS | IS | Yes20 | No | WMH | NS | Yes |
| Kumral et al, 2020 | 2004-2017 | Turkey | Inpatient | 8700/11200  (78%) | 4219/8700  (48%) | 66 (12) | IS | Yes5 | Yes | Acute stroke features, CMB, WMH | 1.5T/3T | Yes |
| Liang et al, 2019 | 2010-2015 | Hong Kong | Inpatient | 451/1821  (25%) | 199/451 (44%) | 66.0 (10.3) | IS | Yes20 | Yes | CMB, cSVD, lacunes, PVS, pre-existing stroke lesions, WMH | 1.5T | Yes |
| Lin et al, 2003 | 1995-1999 | Taiwan | Inpatient | 283/352 (80%) | 95/283 (34%) | 64.4 (8.4) | IS | Yes20 | No | Acute stroke features | 1.5T | Yes |
| Makin et al, 2018 | NS | Scotland | Inpatient/  Outpatient | 151/208  (73%) | 93/151 (62%) | Median=66,  IQR=56-74 | IS^[[15]](#footnote-15)^ | Yes^[[16]](#footnote-16)^ | No | Atrophy, CMB, cSVD, WMH | 1.5T | Yes |
| Mandzia et al, 2016 | 2009-2012 | Canada | Inpatient1 | 92/129  (71%) | 24/92 (26%) | 65.1 (12.0) | IS & TIA^[[17]](#footnote-17)^ | Yes20 | No | CMB, pre-existing stroke lesions | 3T | Yes |
| Molad et al, 2019 | 2008-2014 | Israel | Inpatient | 397/575  (69%) | 171/397 (43%) | 66.9 (9.7) | IS & TIA^[[18]](#footnote-18)^ | No | Yes | CMB, lacunes, PVS, WMH, additional features | 3T | Yes |
| Moulin et al, 2016 | 2004-2009 | France | Inpatient | 188/560  (34%) | NS | Median=67.5,  IQR=55-76 | ICH^[[19]](#footnote-19)^ | Yes^[[20]](#footnote-20)^ | No | Atrophy, CMB, additional features, lacunes, pre-existing stroke lesions, WMH | 1.5T | Yes |
| Schellhorn et al, 2021 | 2015-2017 | Norway | Inpatient | 231/815  (28%) | 101/231 (44%) | 71.8 (11.1) | IS & ICH | No | No | Acute stroke lesions, atrophy, WMH | 1.5T/3T | Yes |
| Schiemanck et al, 2005 | 1999-2001 | Netherlands | Inpatient | 75/115  (65%) | 40/75 (53%) | 63 (15) | IS^[[21]](#footnote-21)^ | Yes5 | Yes | Acute stroke lesions | 0.5T/1.0T/1.5T | Yes |
| Sivakumar et al, 2017 | 2008-2014 | Canada | Inpatient1 | 105/120 (88%) | 36/105 (34%) | NS | IS^[[22]](#footnote-22)^ & TIA | Yes20 | No | Acute stroke features, CMB, WMH | 1.5T | No |
| Sung et al, 2021 | 2015-2018 | Taiwan | Inpatient | 98/112 (88%) | NS | NS | IS | Yes^[[23]](#footnote-23)^ | Yes | Atrophy, cSVD | NS | Yes |
| Xiong et al, 2019 | 2006-2017 | USA | Inpatient | 97/204  (48%) | 43/97 (44%) | 73.92 (8.73) | ICH^[[24]](#footnote-24)^ | Yes20 | No | Acute stroke lesions, atrophy, CMB, cSVD, lacunes, additional stroke features, PVS, WMH | 1.5T | Yes |
| Zhang et al, 2021 | 2015-2018 | China | Inpatient1 | 291/1620^[[25]](#footnote-25)^ | NS | NS | IS & TIA | Yes5 | No | WMH | NS | Yes |
| Zhi et al, 2021 | 2016-2019 | China | Inpatient1 | 157/189  (83%) | 30/157 (19%) | 65.27 (7.37) | IS^[[26]](#footnote-26)^ | Yes5 | Yes | Atrophy, cSVD | 3T | Yes |
| Zhong et al, 2021 | 2018-2019 | China | Inpatient | 103/279  (37%) | 34/103 (33%) | 57.22 (12.95) | IS^[[27]](#footnote-27)^ | Yes5 | No | Atrophy, CMB, WMH | 3T | No |

*Performed adjusted analysis = performed adjusted analysis within the study (i.e. may not have included the imaging feature in the multivariable model if it did not reach statistical significance following univariate analysis).*

***Abbreviations:***

*CMB, cerebral microbleeds; cSVD, cerebral small vessel disease; ICH, intracerebral haemorrhage; IQCODE, Informant Questionnaire on Cognitive Decline in the Elderly; Isch, ischaemic stroke; MMSE, mini-mental state examination; NS, not stated; PSCI, post-stroke cognitive impairment; PSD, post-stroke dementia; PVS, perivascular spaces; SD, standard deviation; TIA, transient ischaemic attack; WMH, white matter hyperintensities;*

# **Supplement 4: Clinical risk factors of stroke population included in cognitive follow-up**

| **Study** | **Age** | **Females** | **Education** | **Hypertension** | **Diabetes** | **Hypercholesterolemia** | **Atrial fibrillation** | **Smoking** | **Alcohol** | **APOE** | **IHD** | **Prior stroke/**  **TIA** | **Stroke severity** | **Proportion with cognitive impairment/dementia prior to stroke** |
| --- | --- | --- | --- | --- | --- | --- | --- | --- | --- | --- | --- | --- | --- | --- |
| Appelros et al, 2005 | NS | NS | NS | NS | NS | NS | NS | NS | NS | NS | NS | NS | NS | NS |
| Banergee et al, 2019 | Mean=73.1,  SD=9.1 | 39% | Educational age (years):  Mean=16.8,  SD=3.5 | 52% | 10% | 44% | 24% | Ex-smoker: 47%,  Current smoker:10% | NS | NS | Heart failure: 4% | Further intracerebral event within 12 months of study entry: 5% | NIHSS:  Median=3.5,  IQR:2-9 | NS |
| Chaudhari et al, 2014 | Mean=59.4,  SD=10.9 | 26% | Median=8 years,  Range=0-18 years | 49% | 33% | 39% | NS | Smoking: 33% | Alcohol: 18% | NS | Ischemic heart disease (angina, myocardial infarction, ischemic cardiomyopathy, EEG abnormality, rhythm disturbances): 15% | Prior stroke: 26% | VCI:  median=7,  range=3-13;  No VCI:  median=5,  range=1-10 | NS |
| Chen et al, 2016 | Mean=63.8,  SD=8.3 | 38% | PSCI:  mean=7.5 years, SD=4.5 years;  No PSCI:  mean=11.2 years,  SD=3.7 years | 82% | 41% | 73% | 4% | Current/former: 52% | Alcohol abuse: 18% | NS | History of myocardial infarction or angina pectoris: 11% | Prior stroke: 21% | PSCI  NIHSS: mean=4.4, SD=3.7;  No PSCI NIHSS: mean=3.2,  SD=3.2 | NS |
| Christ et al, 2019 | CMB+ mean=76.6, SD=8.6;  CMB- mean=76.4,  SD=10.1 | 45% | CMB+ mean=11.6 years, SD=2.8 years;  CMB- mean=11.3 years,  SD=2.7 years; | 82% | 26% | 26% | 35% | Smoking: 12% | NS | NS | NS | NS | CMB+ NIHSS mean=3.9, SD=4.3;  CMB- NIHSS mean=3.2. SD=4.6 | NS |
| Coutureau et al, 2021 | Mean=67.5,  SD=14.1 | 36% | Baccalaureate or higher: 40% | NS | NS | NS | NS | NS | NS | NS | NS | Sequelae of previous stroke: 8% | Median: 4,  IQR: 2-8 | NS |
| Fruhwirth et al, 2020 | Mean=61,  SD=10 | 23% | Mean: 12 years,  SD: 3 years | 74% | 15% | 79% | NS | Active smoking: 39% | NS | NS | NS | NS | NIHSS:  Median=2,  IQR=1-4 | NS |
| Gregoire et al, 2012 | With CMBs: median=65;  Without CMBs: median=62;  range=35-86 | 38% | With CMBs: median=13 years, range=9-24 years;  Without CMBs: median=11 years, range=0-20 years | 65% | 23% | With CMBs:  median cholesterol=5mmol/l, range=3.8-6.8;  Without CMBs:  median cholesterol=5mmol/l, range=1-8.3; | NS | Smoking: 42% | NS | NS | NS | 23% | NS | NS |
| Han et al, 2021 | Range: 40-80 years | 38% | NS | NS | NS | NS | NS | NS | NS | NS | NS | NS | NS | NS |
| Kandiah et al, 2016 A | Mean=61.67,  SD=12.46 | 32% | Mean=4.59 years,  SD=4.46 years | 78% | 41% | 81% | 16% | Smoking history: 29% | NS | NS | IHD: 19% | History of TIA:7%;  History of stroke:15% | NS | NS |
| Kandiah et al, 2016 B | Mean=59.60,  SD=11.18 | 31% | Mean=9.01 years,  SD=3.17 years | 71% | 32% | 98% | 15% | Smoking history: 43% | NS | NS | IHD: 1% | History of TIA:3%;  History of stroke:15% | NIHSS:  mean=3.7,  SD=2.1 | Measured IQCODE but do not explicitly state patients with pre-stroke cognitive impairment were excluded |
| Kang et al, 2013 | NS | NS | NS | NS | NS | NS | NS | NS | NS | NS | NS | NS | NS | NS |
| Kumral et al, 2020 | Mean=66,  SD=12 | 48% | NS | 73% | 29% | 45% | 18% | Current cigarette smoking:18% | NS | APOE e4 allele = 20% | NS | Prior TIA: 5% | NS | NS |
| Liang et al, 2019 | Mean=66.0,  SD=10.3 | 44% | Median=6 years,  IQR=4-9 years | 65% | 25% | 41% | 4% | Current or previous smoker: 35% | NS | NS | 4% | NS | NIHSS:  median=3,  IQR=1-5 | NS |
| Lin et al, 2003 | Mean=64.4,  SD=8.4 | 34% | ≤6y: 62%;  >6y: 38% | 79% | 41% | 27% | NS | NS | NS | NS | Prior heart disease: 30% | Prior stroke: 30% | NIHSS ≤7: 89%;  NIHSS >7: 11% | NS |
| Makin et al, 2018 | Median=66,  IQR=56-74 | 62% | PSCI:  Median=10 years,  IQR=9-10 years;  No PSCI: median=11 years, IQR=10-14 years | 72% | 12% | NS | 9% | Smoker: 28% | Alcohol over recommended limit: 17% | NS | IHD: 19% | Previous stroke: 13% | NIHSS  PSCI:  Median=2,  IQR=1-3;  No PSCI: median=2,  IQR=1-3 | NS |
| Mandzia et al, 2016 | 65.1 (12.0) | 26% | Median: 14 years,  25^th^-75^th^ percentile: 12-16 years | 86% | 17% | NS | 9% | Smoker current: 14% | NS | NS | Previous MI: 12% | NS | NIHSS:  median=1,  25^th^ – 75^th^ percentile=0-2 | NS |
| Molad et al, 2019 | Mean=66.9,  SD=9.7 | 43% | PSCI:  Mean=11.3,  SD=3.9;  No PSCI:  Mean: 13.6,  SD=3.7 | 58% | 27% | 53% | NS | Current smokers: 22% | NS | APOE e4 allele: 18% | NS | NS | PSCI NIHSS: Median=3,  IQR=1-6;  No PSCI NIHSS:  Median=2,  IQR=0-3 | NS |
| Moulin et al, 2016 | NS | NS | NS | NS | NS | NS | NS | NS | NS | NS | NS | NS | NS | NS |
| Schellhorn et al, 2021 | 71.8 (11.1) | 44% | Mean=12.3 years,  SD=3.7 | 47% | 17% | 39% | NS | Previous: 39%;  Current:21% | NS | NS | NS | Previous clinical stroke: 14% | NIHSS score:  Mean=3.7,  SD=4.6 | Mild pre-stroke neurocognitive disorder: 4%;  Major pre-stroke neurocognitive disorder: 4% |
| Schiemanck et al, 2005 | Mean=63,  SD=15 | 53% | NS | NS | NS | NS | NS | NS | NS | NS | NS | NS | NIHSS mean: 11, IQR:7-15 | NS |
| Sivakumar et al, 2017 | Persisting deficits: median=79, IQR=12;  Transient deficits: median=68, IQR=18;  No deficits: median=62, IQR=15 | 34% | NS | 60% | 19% | 50% | NS | NS | NS | NS | NS | Prior stroke/TIA: 25% | Persisting deficits: median=1,  IQR = 2;  Transient deficits: median =1, IQR = 2;  No deficits: median =1, IQR = 2 | NS |
| Sung et al, 2021 | NS | NS | NS | NS | NS | NS | NS | NS | NS | NS | NS | NS | NS | NS |
| Xiong et al, 2019 | Mean=73.92,  SD=8.73 | 44% | Education (score):  Mean:4,  SD:3.4  0: none  1:1-6 years  2: 7-9 years  3:10-13 years  4:14+ years | 71% | 12% | 54% | NS | NS | NS | NS | NS | Prior hemorrhagic stroke: 9% | NS | 11% had pre-existing mild cognitive impairment |
| Zhang et al, 2021 | NS | NS | NS | NS | NS | NS | NS | NS | NS | NS | NS | NS | NS | NS |
| Zhi et al, 2021 | Mean=65.27,  SD=7.37 | 19% | Mean=10.52,  SD=2.90 | 71% | 48% | 16% | NS | Current smoking=63% | Current drinking=26% | NS | NS | NS | NS | NS |
| Zhong et al, 2021 | 57.22 (12.95) | 34/103 (33%) | Illiteracy (<1 year): 5.8%;  Primary (1-6 years): 50%;  Secondary (7-12 years): 37%;  University (>12 years): 8% | 62% | 20% | NS | 11% | Smoking history (≥6 months): 36% | NS | NS | NS | Ischemic stroke history:14% | NIHSS: Median=2,  Qu-Ql=1-4 | IQCODE:  Median=49,  Qu-QL=48-49 |

# **Supplement 5: Cognitive assessment methods**

| **Cognitive assessment methods** | | | |
| --- | --- | --- | --- |
| **Study** | **Developed cognitive outcome**  **N (%)** | **Criteria for cognitive outcome** | **Latest follow-up time** |
| **Post-stroke dementia** | | | |
| Kumral et al, 2020 | 1048/8700 (12%) | DSM-IV and DSM-5 | 3 months |
| Lin et al, 2003 | 26/283 (9%)^[[28]](#footnote-28)^ | ICD-10NA | 3 months |
| Moulin et al, 2016 | 52/188 (28%) | NINDS-AIREN | Median=6 years |
| Xiong et al, 2019 | 25/97 (26%) | National institute on aging and Alzheimer’s Association workgroup criteria^47^ | Median=2.5 years,  IQR=1.5-3.8 years |
| **Post-stroke cognitive impairment** | | | |
| Appelros et al, 2005 | 9/56 (16%) | MMSE <24 | 5 years |
| Banergee et al, 2019 | 60/117 (51%) | MoCA <26 | 12 months |
| Chaudhari et al, 2014 | 46/102 (48%)^[[29]](#footnote-29)^ | VCI-ND: criteria of the Canadian Study of Health and Aging^48^;  VaD: NINDS-AIREN | 6 months |
| Chen et al, 2016 | 31/56 (55%) | Chinese version of NINDS-CSN neuropsychological protocol | 3 to 12 months  (Mean=7.1 months,  SD=2.3 months) |
| Christ et al, 2019 | NS | CERAD-plus | 6 months |
| Coutureau et al, 2021 | 167/348 (48%) | MoCA <26 | 3 months |
| Fruhwirth et al, 2020 | 18/82 (22%) | Neuropsychological battery | 15 months  Mean=15.9 months,  SD=1.9 months |
| Gregoire et al, 2012 | NS | Neuropsychological battery  (study focuses on frontal-executive impairment) | Mean=5.67 years,  Range=4.75-7.08 years |
| Han et al, 2021 | 124/208 (60%) | MoCA <26 | 3 months |
| Kandiah et al, 2016 A | 78/209 (37%) | MMSE ≤25 or MoCA ≤22 | 3-6 months  Mean = 3.66 months,  SD=3.24 months |
| Kandiah et al, 2016 B | 35/185 (19%) | MMSE ≤25 or MoCA ≤22 | 3-6 months^[[30]](#footnote-30)^ |
| Kang et al, 2013 | NS | MMSE | 1 year |
| Liang et al, 2019 | NS | Hong Kong version of the MMSE ≤26 | 15 months |
| Makin et al, 2018 | 29/151 (19%) | ACE-R <82 | 12 months |
| Mandzia et al, 2016 | Executive function: 15/92 (16%);  Psychomotor processing speed: 16/92 (17%);  Memory: 6/92 (7%) | Modified version of the Canadian Stroke Network and National Institute on Neurological Disorders and Stroke vascular cognitive impairment battery  (study focuses on executive function, psychomotor processing speed, memory) | 3 months |
| Molad et al, 2019 | 80/397 (20%) | MoCA and NeuroTrax computerized cognitive testing.  PSCI: modified Petersen criteria;  PSD: DSM IV-TR | 24 months |
| Schellhorn et al, 2021 | 128/231 (55%)^[[31]](#footnote-31)^ | DSM-5 | 3 months |
| Schiemanck et al, 2005 | NS | MMSE | 1 year |
| Sivakumar et al, 2017 | 19/105 (18%)^[[32]](#footnote-32)^ | MoCA <26 | 3 months |
| Sung et al, 2021 | 7 days: NS/112  3 months: NS/112  1 year: NS/98 | MoCA | 7 days, 3 months, 1 year  (study assessed cognitive performance over time) |
| Zhang et al, 2021 | NS | MoCA | 12 months |
| Zhi et al, 2021 | 95/157 (61%) | Neuropsychological battery and MMSE^[[33]](#footnote-33)^ | 3 months |
| Zhong et al, 2021 | 55/103 (53%) | Modified version of the vascular dementia battery | 3 months |

**Abbreviations:** *AIREN, Association Internationale pour la Recherche et l’Enseignement en Neurosciences; CERAD, Consortium to Establish a Registry for Alzheimer's Disease; CSN, Canadian Stroke Network; DSM-5, Diagnostic and Statistical Manual of Mental Disorders 5; DSM-IV, Diagnostic and Statistical Manual of Mental Disorders 4; DSM-IV-TR, Diagnostic and Statistical Manual of Mental Disorders 4 text revision; ICD-10NA, International Classification of Diseases tenth revision: neurological adaptation; IQR, inter-quartile range; MMSE, Mini-Mental State Exam; MoCA, Montreal Cognitive Assessment; NINDS, National Institute of Neurological Disorders and Stroke (NINDS); PSCI, post-stroke cognitive impairment; PSD, post-stroke dementia; VaD, vascular dementia; VCI-ND, vascular cognitive impairment no dementia.*

# **Supplement 6: Atrophy**

**eTable 6.1: PRESENCE of cerebral atrophy and post-stroke DEMENTIA**

|  | | | | | | **Unadjusted analysis** | | | | **Adjusted analysis** | | | |
| --- | --- | --- | --- | --- | --- | --- | --- | --- | --- | --- | --- | --- | --- |
| **Study** | **Neuroimaging feature** | **MRI sequence** | **Measuring technique** | **Developed**  **PSD** | **Not developed PSD** | **Effect size** | **Sig. level** | **Statistical test** | **Associated with PSD** | **Effect size** | **Sig. level** | **Statistical test** | **Associated with PSD** |
| Moulin et al, 2016 | Presence of global cortical atrophy | NS | 4 point rating scale^49^  (Presence defined as 1 point or more) | 96% | 75% | OR=5.36,  95%CI=1.22-23.55§ | 0.03 | Logistic regression  (Calculated by review authors) | Yes* | N/A | N/A | N/A | N/A |

*finding visualised in the harvest plot (Figure 2)

§data included in the meta-analysis

Abbreviations: N/A, not applicable, NS, not stated

**eTable 6.2: PRESENCE of cerebral atrophy and post-stroke COGNITIVE IMPAIRMENT**

|  | | | | | | **Unadjusted analysis** | | | | **Adjusted analysis** | | | |
| --- | --- | --- | --- | --- | --- | --- | --- | --- | --- | --- | --- | --- | --- |
| **Study** | **Neuroimaging feature** | **MRI sequence** | **Measuring technique** | **Developed**  **PSCI** | **Not developed PSCI** | **Effect size** | **Sig. level** | **Statistical test** | **Associated with PSCI** | **Effect size** | **Sig. level** | **Statistical test** | **Associated with PSCI** |
| Chen et al, 2016 | Presence of global cortical atrophy | NS | 4-point rating scale^49^  (Presence defined as 1 point or more) | 48% | 28% | Cohen’s d=0.48 | 0.120 | Chi square test | No* | OR=5.730,  95% CI= 1.128-29.101 | 0.035 | Multivariate logistic regression | Yes* |
| Chen et al, 2016 | Presence of global cortical atrophy | NS | 4-point rating scale^49^  (Presence defined as 1 point or more) | 48% | 28% | OR=2.41,  95%CI=0.79-7.4§ | 0.12 | Logistic regression  (Calculated by review authors) | No | N/A | N/A | N/A | N/A |
| Kandiah et al, 2016 A | Presence of global cortical atrophy | T1 | Modified version of 4-point rating scale^49^  (Presence defined as 1 point or more) | 47% | 30% | OR=2.05,  95% CI=1.15-3.66§ | 0.01 | Logistic regression  (Calculated by review authors) | Yes* | N/A | N/A | N/A | N/A |

*finding visualised in the harvest plot (Figure 2)

§data included in the meta-analysis

Abbreviations: N/A, not applicable, NS, not stated

**eTable 6.3 SEVERITY of cerebral atrophy and post-stroke DEMENTIA**

|  | | | | | | **Unadjusted analysis** | | | | **Adjusted analysis** | | | |
| --- | --- | --- | --- | --- | --- | --- | --- | --- | --- | --- | --- | --- | --- |
| **Study** | **Neuroimaging feature** | **MRI sequence** | **Measuring technique** | **Developed**  **PSD** | **Not developed PSD** | **Effect size** | **Sig. level** | **Statistical test** | **Associated with PSD** | **Effect size** | **Sig. level** | **Statistical test** | **Associated with PSD** |
| Moulin et al, 2016 | Cortical atrophy score per 1-point increase | NS | 4 point rating scale^49^ | 0: 4%;  1: 21%;  2&3: 75% | 0: 25%;  1: 41%;  2&3: 34% | N/A | N/A | N/A | N/A | SHR=2.61, 95% CI=1.70-4.01 | <0.0001 | Backward stepwise multivariable analysis | Yes* |
| Moulin et al, 2016 | Severity of global cortical atrophy | NS | 4 point rating scale^49^ | 0: 4%;  1: 21%;  2&3: 75% | 0: 25%;  1: 41%;  2&3: 34% | N/A | N/A | N/A | N/A | 0:  SHR=1.98, 95% CI=0.40-9.73;  1:  SHR=4.78, 95% CI=0.96-23.96;  2&3:  SHR=7.24, 95% CI=1.10-47.74;  When using score as an ordinal variable:  SHR=2.02, 95% CI=1.28-3.19 | 0:  0.40;  1:  0.057;  2&3:  0.040;  When using score as an ordinal variable: 0.002 | Fine-Gray model | Yes |
| Xiong et al, 2019 | Global cortical atrophy score | T1 | 4 point rating scale^49^ | NS | NS | HR=1.049;  95% CI=0.945-1.164 | 0.368 | Univariable Cox proportional hazards analysis | No* | N/A | N/A | N/A | N/A |

*finding visualised in the harvest plot (Figure 2)

§data included in the meta-analysis

Abbreviations: N/A, not applicable, NS, not stated

**eTable 6.4: SEVERITY of cerebral atrophy and post-stroke COGNITIVE IMPAIRMENT**

|  | | | | | | **Unadjusted analysis** | | | | **Adjusted analysis** | | | |
| --- | --- | --- | --- | --- | --- | --- | --- | --- | --- | --- | --- | --- | --- |
| **Study** | **Neuroimaging feature** | **MRI sequence** | **Measuring technique** | **Developed**  **PSCI** | **Not developed PSCI** | **Effect size** | **Sig. level** | **Statistical test** | **Associated with PSCI** | **Effect size** | **Sig. level** | **Statistical test** | **Associated with PSCI** |
| Kandiah et al, 2016 A | Global cortical atrophy stages | T1 | Modified version of 4-point rating scale^49^ | 0=53%,  1=38%,  2=9%,  3=0 | 0=69%,  1=27%,  2=3%,  3=0 | NS | 0.026 | X^2^ test or Fisher’s Exact test | Yes | β coefficient = 0.191  SE=0.325  (scoring 0,1,2) | 0.556 | Multivariate logistic regression | No |
| Kandiah et al, 2016 A | Global cortical atrophy score | T1 | Modified version of 4-point rating scale^49^ | Mean=0.56,  SD=0.66 | Mean=0.34,  SD=0.54 | NS | 0.009 | Independent sample t-test or Wilcoxon-Mann-Whitney test | Yes* | N/A | N/A | N/A | N/A |
| Kandiah et al, 2016 B | Global cortical atrophy score | T1 | Modified version of 4-point rating scale^49^ | Mean=0.94,  SD=0.60 | Mean=0.54,  SD=0.58 | NS | <0.001 | Independent sample t-test or Wilcoxon-Mann-Whitney test | Yes* | N/A | N/A | N/A | N/A |
| Makin et al, 2018 | Total atrophy score | NS | NS | Median=6,  IQR=3-6 | Median=4,  IQR=2-6 | OR= 1.15,  95% CI=0.96-1.37 | 0.13 | Logistic regression | No* | OR=1.06,  95% CI=0.79-1.41 | 0.68 | Logistic regression | No* |

*finding visualised in the harvest plot (Figure 2)

§data included in the meta-analysis

Abbreviations: MTA, medial temporal lobe atrophy; N/A, not applicable; NS, not stated

**eTable 6.5: PRESENCE of LOCALISED atrophy and post-stroke COGNITIVE IMPAIRMENT**

|  | | | | | | **Unadjusted analysis** | | | | **Adjusted analysis** | | | |
| --- | --- | --- | --- | --- | --- | --- | --- | --- | --- | --- | --- | --- | --- |
| **Study** | **Neuroimaging feature** | **MRI sequence** | **Measuring technique** | **Developed**  **PSCI** | **Not developed PSCI** | **Effect size** | **Sig. level** | **Statistical test** | **Associated with PSCI** | **Effect size** | **Sig. level** | **Statistical test** | **Associated with PSCI** |
| Chen et al, 2016 | Presence of MTA | NS | 5-point rating scale^50^  (MTA defined as ≥2 points) | 13% | 20% | Cohen’s d=0.19 | 0.360 | Chi square test | No* | NS | NS | Multivariate logistic regression | No* |
| Schellhorn et al, 2021 | Presence of MTA | NS | 5-point rating scale^50^  (‘MTA of both sides was ≥1.5 under the age of 75, a value  ≥2 below the age of 85, and a value of ≥2.5 below 95 years’) | 42% | 27% | N/A | N/A | N/A | N/A | OR=1.82,  95% CI=0.94-3.52 | 0.074 | Multiple logistic regression | No* |
| Sung et al, 2021 | Presence of hippocampal atrophy | NS | 5-point rating scale^50^  (score≥3 for patients younger than 75  years old and≥2 for patients over 75 years) | NS | NS | N/A | N/A | N/A | N/A | No: REF;  Yes:  B=-1.89,  SE=1.41 | 0.180 | Generalised estimating equation model (multivariable model) | No* |

*finding visualised in the harvest plot (Figure 2)

§data included in the meta-analysis

Abbreviations: MTA, medial temporal lobe atrophy; N/A, not applicable; NS, not stated

**eTable 6.6: SEVERITY of LOCALISED atrophy and post-stroke COGNITIVE IMPAIRMENT**

|  | | | | | | **Unadjusted analysis** | | | | **Adjusted analysis** | | | |
| --- | --- | --- | --- | --- | --- | --- | --- | --- | --- | --- | --- | --- | --- |
| **Study** | **Neuroimaging feature** | **MRI sequence** | **Measuring technique** | **Developed**  **PSCI** | **Not developed PSCI** | **Effect size** | **Sig. level** | **Statistical test** | **Associated with PSCI** | **Effect size** | **Sig. level** | **Statistical test** | **Associated with PSCI** |
| Zhi et al, 2021 | MTA score | NS | 5-point rating scale^50^ | Mean=0.59,  SD=0.75 | Mean=0.51,  SD=0.66 | NS | 0.251 | Independent t-test | No* | N/A | N/A | N/A | N/A |
| Zhong et al, 2021 | MTA score | NS | 5-point rating scale^50^ | Median=2,  Qu-Ql=1-4 | Median=0,  Qu-Ql=0-2 | t/z/x2=-3.596 | <0.001 | Mann Whitney U | Yes* | N/A | N/A | N/A | N/A |

*finding visualised in the harvest plot (Figure 2)

§data included in the meta-analysis

Abbreviations: N/A, not applicable, NS, not stated

# **Supplement 7: White matter hyperintensities**

**eTable 7.1: PRESENCE of WMH and post-stroke DEMENTIA**

|  | | | | | | **Unadjusted analysis** | | | | **Adjusted analysis** | | | |
| --- | --- | --- | --- | --- | --- | --- | --- | --- | --- | --- | --- | --- | --- |
| **Study** | **Neuroimaging feature** | **MRI sequence** | **Measuring technique** | **Developed**  **PSD** | **Not developed PSD** | **Effect size** | **Sig. level** | **Statistical test** | **Associated with PSD** | **Effect size** | **Sig. level** | **Statistical test** | **Associated with PSD** |
| Kumral et al, 2020 | Presence of WMH | FLAIR | Fazekas scale^51^  (Presence: score 1-3) | 58% | 54% | NS | 0.002 | Chi-square | Yes* | N/A | N/A | N/A | N/A |
| Kumral et al, 2020 | Presence of WMH | FLAIR | Fazekas scale^51^  (Presence: score 1-3) | 58% | 54% | OR=1.22,  95% CI=1.07-1.37§ | <0.01 | Logistic regression  (Calculated by review authors) | Yes | N/A | N/A | N/A | N/A |
| Moulin et al, 2016 | Presence of WMH | NS | Fazekas scale^51^ | 0: 4%;  1: 15%;  2: 35%;  3: 46% | 0: 18%;  1: 32%;  2: 37%;  3: 14% | OR=5.36,  95% CI=1.22-23.55§ | 0.03 | Logistic regression  (Calculated by review authors) | Yes* | N/A | N/A | N/A | N/A |

*finding visualised in the harvest plot (Figure 2)

§data included in the meta-analysis

Abbreviations: N/A, not applicable, NS, not stated

**eTable 7.2: PRESENCE of WMH and post-stroke COGNITIVE IMPAIRMENT**

|  | | | | | | **Unadjusted analysis** | | | | **Adjusted analysis** | | | |
| --- | --- | --- | --- | --- | --- | --- | --- | --- | --- | --- | --- | --- | --- |
| **Study** | **Neuroimaging feature** | **MRI sequence** | **Measuring technique** | **Developed**  **PSCI** | **Not developed PSCI** | **Effect size** | **Sig. level** | **Statistical test** | **Associated with PSCI** | **Effect size** | **Sig. level** | **Statistical test** | **Associated with PSCI** |
| Schellhorn et al, 2021 | Presence of WMH | NS | Fazekas scale^51^  (Grade 1=normal,  Grade 2= normal for patients >71 years,  Grade 3=always pathological) | 49% | 26% | N/A | N/A | N/A | N/A | OR=2.18,  95% CI=1.14-4.17 | 0.031 | Multiple logistic regression | Yes* |
| Sivakumar et al, 2017 | Presence of WMH | FLAIR | Modified Fazekas scale^52^  (MRI performed on day 30 after stroke) | 79% | Transient deficits: 66%;  No deficits:48% | X^2^=8.12 | 0.017 | Logistic regression  (study compared three cognitive groups) | Yes* | N/A | N/A | N/A | N/A |
| Sivakumar et al, 2017 | Presence of WMH | FLAIR | Modified Fazekas scale^52^  (MRI performed on day 30 after stroke) | 79% | 56% | OR=4.22,  95% CI=1.15-15.56§ | 0.03 | Logistic regression  (Calculated by review authors) | Yes | N/A | N/A | N/A | N/A |

*finding visualised in the harvest plot (Figure 2)

§data included in the meta-analysis

Abbreviations: N/A, not applicable, NS, not stated

**eTable 7.3: SEVERITY of WMH and post-stroke DEMENTIA**

|  | | | | | | **Unadjusted analysis** | | | | **Adjusted analysis** | | | |
| --- | --- | --- | --- | --- | --- | --- | --- | --- | --- | --- | --- | --- | --- |
| **Study** | **Neuroimaging feature** | **MRI sequence** | **Measuring technique** | **Developed**  **PSD** | **Not developed PSD** | **Effect size** | **Sig. level** | **Statistical test** | **Associated with PSD** | **Effect size** | **Sig. level** | **Statistical test** | **Associated with PSD** |
| Kumral et al, 2020 | WMH score | FLAIR | Fazekas scale^51^ | NS | NS | 1:  OR=1.23,  95%CI=1.06-1.43;  2:  OR=5.39, 95%CI=4.45-6.52;  3:  OR=20.94, 95%CI=11.94-36.72 | 1:  0.006  2:  0.0001  3:  0.0001 | Logistic regression | Yes* | 1:  OR=0.94, 95%CI=0.80-1.12;  2:  OR=3.43, 95%CI=2.75-4.27;  3:  OR=3.43, 95%CI=2.75-4.27 | 1:  0.48;  2:  0.0001;  3:  0.0001 | Multiple logistic regression | Yes* |
| Moulin et al, 2016 | Severity of WMH | NS | Fazekas scale^51^ | 0: 4%;  1: 15%;  2: 35%;  3: 46% | 0: 18%;  1: 32%;  2: 37%;  3: 14% | N/A | N/A | N/A | N/A | 0:  reference;  1: SHR=1.37, 95%CI=0.30-6.31;  2: SHR=1.66, 95%CI=0.36-7.55;  3:  SHR=4.09, 95% CI=0.87-19.15;  When using score as an ordinal variable:  SHR=1.80, CI=1.17-2.75 | 0: reference;  1:  0.69;  2:  0.51;  3:  0.074;  When using score as an ordinal variable:  0.007 | Fine-Gray model | Yes* |
| Xiong et al, 2019 | Severity of WMH | NS | Fazekas score^51^ | NS | NS | HR=1.509,  95% CI=1.104-2.063 | 0.010 | Univariable Cox proportional hazards analysis | Yes* | HR=1.427,  95% CI=1.057-1.927 | 0.02 | Multivariable Cox proportional hazards | Yes* |

*finding visualised in the harvest plot (Figure 2)

§data included in the meta-analysis

Abbreviations: N/A, not applicable, NS, not stated

**eTable 7.4: SEVERITY of WMH and post-stroke COGNITIVE IMPAIRMENT**

|  | | | | | | **Unadjusted analysis** | | | | **Adjusted analysis** | | | |
| --- | --- | --- | --- | --- | --- | --- | --- | --- | --- | --- | --- | --- | --- |
| **Study** | **Neuroimaging feature** | **MRI sequence** | **Measuring technique** | **Developed**  **PSCI** | **Not developed PSCI** | **Effect size** | **Sig. level** | **Statistical test** | **Associated with PSCI** | **Effect size** | **Sig. level** | **Statistical test** | **Associated with PSCI** |
| Appelros et al, 2005 | WMH score | T2 | 4-point rating scale^53^ | NS | NS | Correlation coefficient = 0.44 | <0.01 level (two-tailed) | Spearman’s correlation | Yes* | N/A | N/A | N/A | N/A |
| Appelros et al, 2005 | Basal ganglia score | T2 | 4-point scale^53^ | NS | NS | Correlation coefficient = 0.12 | Not significant | Spearmans correlation | No | N/A | N/A | N/A | N/A |
| Banergee et al, 2019 | Grades of periventricular WMH | T2 & FLAIR | Fazekas scale^51^ | IQR=0-1 | IQR=0-0 | NS | 0.0545 | Independent  t-test or Mann–Whitney U test | No* | OR=1.15,  95% CI=0.54-2.44 | 0.725 | Multivariate logistic regression | No* |
| Chaudhari et al, 2014 | ARWMC score | T2 & FLAIR | 4-point rating scale^53^ | Median=4.5,  Range=0-15 | Median=1.5,  Range=0-12 | NS | 0.001 | Mann Whitney U | Yes* | OR= 1.332,  95% CI=1.082-1.640§ | 0.007 | Binary logistic regression | Yes* |
| Chen et al, 2016 | ARWMC score | FLAIR & T2 | 4-point rating scale^53^ | Mean=6.6,  SD=4.0 | Mean=5.6,  SD=3.8 | Cohen’s d=0.26 | 0.338 | Independent sample t test | No* | NS | NS | Multivariate logistic regression | No* |
| Fruhwirth et al, 2020 | Severity of deep WMH | NS | Fazekas scale^51^  (Merged scores 2&3) | NS | NS | NS | NS | ANOVA | Yes | N/A | NS | ANCOVA | No |
| Fruhwirth et al, 2020 | Severity of periventricular WMH | NS | Fazekas scale^51^  (Merged scores 2&3) | NS | NS | NS | NS | ANOVA | No* | N/A | NS | ANCOVA | No* |
| Gregoire et al, 2012 | WMH score | T2/FLAIR | 4-point rating scale^53^ | NS | NS | Frontal executive functions: OR = 1.11,  95% CI = 0.93-1.33 | 0.247 | Unadjusted binary logistic regression | No  (frontal-executive impairment) | N/A | N/A | N/A | N/A |
| Han et al, 2021 | WMH score | T1/T2/FLAIR | Fazekas scale^51^ | NS | NS | OR is visualised in a figure | <0.05 | Single factor binary logistic regression | Yes* | OR is visualised in a figure | <0.05 | Multivariate binary logistic regression analysis | Yes* |
| Kandiah et al, 2016 A | WMH score | T2 | Fazekas scale^51^ | Mean=2.01,  SD=0.80 | Mean=1.31,  SD=0.96 | NS | <0.001 | Independent sample t-test or Wilcoxon-Mann-Whitney test | Yes* | N/A | N/A | N/A | N/A |
| Kandiah et al, 2016 A | WMH score | T2 | Fazekas scale^51^ | 0-1=28%;  2=41%;  3=31%; | 0-1=65%;  2=20%;  3=15%; | NS | <0.001 | X^2^ test or Fisher’s Exact test | Yes | β coefficient = 0.288  SE=0.222 | P=0.194  95% CI=-0.15-0.75  (scoring 0,1,2) | Multivariate logistic regression | No |
| Kandiah et al, 2016 B | WMH score | T2 | Fazekas scale^51^ | Mean=2.37,  SD=0.69 | Mean=1.67,  SD=0.73 | NS | <0.001 | Independent sample t-test or Wilcoxon-Mann-Whitney test | Yes* | N/A | N/A | N/A | N/A |
| Kang et al, 2013 | PVWMHs | FLAIR | Fazekas scale^51^ categorised into: Mild (grades 1&2) and severe (grades 2&3) | NS | NS | N/A | N/A | N/A | N/A | NS | NS | Repeated measures ANOVA | Yes* |
| Kang et al, 2013 | DWMHs | FLAIR | Fazekas scale^51^ categorised into: Mild (grades 1&2) and severe (grades 2&3) | NS | NS | N/A | N/A | N/A | N/A | NS | NS | Repeated measures ANOVA | Yes |
| Liang et al, 2019 | WMH score | NS | Fazekas scale^51^ | NS | NS | β=-0.46,  SE=0.08 | <0.001 | Univariate linear mixed model | Yes* | OR = 1.15,  CI = 1.02-1.26§ | 0.006 | Generalised estimating equation model | Yes* |
| Makin et al, 2018 | WMH score | NS | 4-point rating scale^53^ | Median = 4,  IQR = 2-6 | Median = 2,  IQR = 2-4 | OR=1.42,  95% CI = 1.11-1.83 | 0.006 | Logistic regression | Yes* | OR = 1.58  95% CI = 1.05-2.44§ | 0.03 | Logistic regression | Yes* |
| Makin et al, 2018 | Periventricular space score | NS | Defined according to STRIVE criteria^54^ | Median = 5,  IQR = 5-7 | Median = 5,  IQR = 4-6 | OR = 1.12  95% CI = 0.93-1.34 | 0.22 | Logistic regression | No | OR = 1.15  95% CI = 0.91-1.44 | 0.24 | Logistic regression | No |
| Molad et al, 2019 | WMH score | NS | Fazekas scale^51^ | Mean=0.6,  SD=0.5 | Mean=0.4,  SD=0.5 | NS | 0.012 | Independent t-test | Yes* | N/A | N/A | N/A | N/A |
| Sivakumar et al, 2017 | Severity of WMH | FLAIR | Fazekas scale^51^  (MRI performed day 30 after stroke) | 0: 16%;  1: 58%;  2: 16%;  3: 5%  (N=1 missing) | 0:  Transient deficits=34%,  No deficits=52%;  1:  Transient deficits=52%,  No deficits=46%;  2:  Transient deficits=13%,  No deficits=2%;  3:  Transient deficits=0%,  No deficits=0% | NS | 0: 0.017;  1: 0.637;  2: 0.048;  3: 0.102 | Logistic regression  (study compared three cognitive groups) | Yes*  (score 2) | N/A | N/A | N/A | N/A |
| Zhang et al, 2021 | Severity of WMH | DWI | Fazekas scale^51^ | NS | NS | NS | NS | Univariable ordinal regression | NS | NS | NS | Multivariable ordinal regression | No* |
| Zhong et al, 2021 | PVH score | FLAIR | Fazekas scale^51^ | Median=2,  Qu-Ql=1-2 | Median=1,  Qu-Ql=0-1 | t/z/x2=-2.997 | 0.003 | Mann Whitney U | Yes* | N/A | N/A | N/A | N/A |
| Zhong et al, 2021 | DWMH score | FLAIR | Fazekas scale^51^ | Median=1,  Qu-Ql=1-2 | Median=0,  Qu-Ql=0-1 | t/z/x2=-2.769 | 0.006 | Mann Whitney U | Yes | N/A | N/A | N/A | N/A |

*finding visualised in the harvest plot (Figure 2)

§data included in the meta-analysis

3 studies reported both deep and periventricular WMH, we included periventricular WMH in the harvest plot as it is more representative of total WMH.

Abbreviations: N/A, not applicable, NS, not stated

# **Supplement 8: Cerebral microbleeds**

**eTable 8.1: PRESENCE of cerebral microbleeds and post-stroke DEMENTIA**

|  | | | | | | **Unadjusted analysis** | | | | **Adjusted analysis** | | | |
| --- | --- | --- | --- | --- | --- | --- | --- | --- | --- | --- | --- | --- | --- |
| **Study** | **Neuroimaging feature** | **MRI sequence** | **Measuring technique** | **Developed**  **PSD** | **Not developed PSD** | **Effect size** | **Sig. level** | **Statistical test** | **Associated with PSD** | **Effect size** | **Sig. level** | **Statistical test** | **Associated with PSD** |
| Kumral et al, 2020 | Presence of CMBs | T2* | Brain Observer MicroBleed Scale criteria^55^ | 13% | 9% | OR=1.47, 95%CI=1.21-1.78 | 0.0001 | Logistic regression | Yes* | OR=1.31, 95%CI=1.06-1.63§ | 0.01 | Multiple logistic regression | Yes* |
| Moulin et al, 2016 | Presence of >5 CMBs | NS | Counted and categorised as either ≤5mm or >5mm | 42% | 18% | OR=3.42,  95% CI=1.69-6.93 | <0.01 | Logistic regression  (Calculated by review authors) | Yes* | SHR=2.33, 95% CI=1.38-3.94 | <0.0001 | Backward stepwise multivariable analysis | Yes* |
| Xiong et al, 2019 | Presence of lobar CMBs ≥5 | GRE | NS | NS | NS | HR=2.051,  95% CI=0.908-4.631 | 0.084 | Univariable Cox proportional hazards analysis | No* | HR=1.105,  95% CI=0.431-2.834 | 0.835 | Multivariable Cox proportional hazards regression | No* |

*finding visualised in the harvest plot (Figure 2)

§data included in the meta-analysis

Abbreviations: N/A, not applicable, NS, not stated

**eTable 8.2: PRESENCE of cerebral microbleeds and post-stroke COGNITIVE IMPAIRMENT**

|  | | | | | | **Unadjusted analysis** | | | | **Adjusted analysis** | | | |
| --- | --- | --- | --- | --- | --- | --- | --- | --- | --- | --- | --- | --- | --- |
| **Study** | **Neuroimaging feature** | **MRI sequence** | **Measuring technique** | **Developed**  **PSCI** | **Not developed PSCI** | **Effect size** | **Sig. level** | **Statistical test** | **Associated with PSCI** | **Effect size** | **Sig. level** | **Statistical test** | **Associated with PSCI** |
| Christ et al, 2019 | Presence of CMBs | T2*-GRE or susceptibility-weighted images (SWI)-MRI scans | ‘small circular or rounded, hypointense lesions ranging from 2 to 10 mm in size’ | NS | NS | N/A | N/A | N/A | N/A | NS | MMSE: 0.024^[[34]](#footnote-34)^ | Transformed CERAD-plus variables to z-scores and adjusted significance using Bonferroni’s correction | Yes* |
| Fruhwirth et al, 2020 | Presence of ≥1 CMBs | NS | Presence vs. absence | NS | NS | NS | N/A | N/A | N/A | β=-0.07 | 0.549 | Multiple linear regression | No* |
| Gregoire et al, 2012 | Presence of CMBs | T2*- GRE | The Microbleed Anatomical Rating Scale^56^ | 58% | 17% | Frontal executive functions:  OR = 8.40,  95% CI = 1.27-55.39 | Frontal executive functions: 0.02734 | Unadjusted binary logistic regression | Yes  (Frontal-executive impairment) | N/A | N/A | N/A | N/A |
| Liang et al, 2019 | Presence of CMBs | NS | NS | NS | NS | N/A | N/A | N/A | N/A | OR = 1.61,  95% CI = 0.93-2.77§ | 0.09 | Generalised estimating equation model | No* |
| Mandzia et al, 2016 | Presence of CMBs | NS | NS | NS | NS | Executive function: NS  Psychomotor Processing Speed: NS  Memory: NS | NS | Depending on the variable:  t test/  ANOVA/Wilcoxon Rank-sum test/ Fisher exact test/Spearman correlation | No | N/A | N/A | N/A | N/A |
| Molad et al, 2019 | Presence of lobar CMBs^[[35]](#footnote-35)^ | SWI/T1/DWI | ‘round hypointense lesions  on SWI with a diameter <10 mm’ | 20% | 11% | HR=1.98,  95% CI=1.04-3.79 | 0.039 | Logistic regression | Yes* | HR=1.83,  95% CI=0.84-3.99 | 0.127 | Multivariate Cox regression | No* |
| Molad et al, 2019 | Deep Microbleeds yes/no | SWI/T1/DWI | ‘round hypointense lesions  on SWI with a diameter <10 mm’ | 6% | 4% | HR=1.46,  95% CI=1.45-4.18 | 0.483 | Logistic regression | No | N/A | N/A | N/A | N/A |
| Sivakumar et al, 2017 | Presence of CMBs | GRE | Manually identified | 16% | Transient deficits:13%;  No deficits:6% | NS | 0.738 | Logistic regression  (study compares three cognitive groups) | No* | N/A | N/A | N/A | N/A |
| Zhong et al, 2021 | Presence of CMBs | SWI | ‘small (2–  10mm) hypointense lesions with a clear margin’ | 42% | 31% | X2=1.130 | 0.288 | Chi-squared test | No* | N/A | N/A | N/A | N/A |

*finding visualised in the harvest plot (Figure 2)

§data included in the meta-analysis

Abbreviations: N/A, not applicable, NS, not stated

**eTable 8.3: NUMBER of cerebral microbleeds and post-stroke DEMENTIA**

|  | | | | | | **Unadjusted analysis** | | | | **Adjusted analysis** | | | |
| --- | --- | --- | --- | --- | --- | --- | --- | --- | --- | --- | --- | --- | --- |
| **Study** | **Neuroimaging feature** | **MRI sequence** | **Measuring technique** | **Developed**  **PSD** | **Not developed PSD** | **Effect size** | **Sig. level** | **Statistical test** | **Associated with PSD** | **Effect size** | **Sig. level** | **Statistical test** | **Associated with PSD** |
| **Xiong et al, 2019** | Number of CMBs | GRE | NS | NS | NS | HR=0.997,  95% CI=0.990-1.005 | 0.521 | Univariable Cox proportional hazards analysis | No* | N/A | N/A | N/A | N/A |

*finding visualised in the harvest plot (Figure 2)

§data included in the meta-analysis

Abbreviations: N/A, not applicable, NS, not stated

**eTable 8.4: NUMBER of cerebral microbleeds and post-stroke COGNITIVE IMPAIRMENT**

|  | | | | | | **Unadjusted analysis** | | | | | **Adjusted analysis** | | | |
| --- | --- | --- | --- | --- | --- | --- | --- | --- | --- | --- | --- | --- | --- | --- |
| **Study** | **Neuroimaging feature** | **MRI sequence** | **Measuring technique** | **Developed**  **PSCI** | **Not developed PSCI** | **Effect size** | **Sig. level** | **Statistical test** | | **Associated with PSCI** | **Effect size** | **Sig. level** | **Statistical test** | **Associated with PSCI** |
| **Christ et al, 2019** | Number of CMBs | T2*-GRE or susceptibility-weighted images (SWI)-MRI scans | ‘small circular or rounded, hypointense lesions ranging from 2 to 10 mm in size’ | NS | NS | N/A | N/A | N/A | | N/A | Mean Pearson’s r=-0.22 | NS | Transformed CERAD-plus variables to z-scores and adjusted significance using Bonferroni’s correction | Yes* |
| **Christ et al, 2019** | Multilocular CMBs | T2*-GRE or susceptibility-weighted images (SWI)-MRI scans | ‘small circular or rounded, hypointense lesions ranging from 2 to 10 mm in size’  Multiocular: more than one CMB in either ‘lobar’, ‘cerebellar’, ‘deep’ brain areas | NS | NS | N/A | N/A | N/A | | N/A | NS | MMSE: 0.020  Table 2 in the study outlines the association between presence/absence of CMBs and other neuropsychological sub-tests. | Transformed CERAD-plus variables to z-scores and adjusted significance using Bonferroni’s correction | Yes |
| Han et al, 2021 | Number of CMB | SWI/T2*GRE | ≥1 CMB | NS | NS | OR is visualised in a figure. | <0.05 | Single factor binary logistic regression | Yes* | | OR is visualised in a figure. | NS | Multivariate binary logistic regression analysis  (adjusted for other neuroimaging features) | No* |
| Kandiah et al, 2016 A | Number of CMBs: Cortical | GRE | The Microbleed Anatomical Rating Scale^56^ | Mean=0.68,  SD=2.60 | Mean=0.67,  SD=3.02 | NS | 0.127 | Independent sample t-test or Wilcoxon-Mann-Whitney test | | No | N/A | N/A | N/A | N/A |
| Kandiah et al, 2016 A | Number of CMBs:  Sublobar | GRE | The Microbleed Anatomical Rating Scale^56^ | Mean=0.78,  SD=2.61 | Mean=0.36,  SD=1.14 | NS | 0.199 | Independent sample t-test or Wilcoxon-Mann-Whitney test | | No | N/A | N/A | N/A | N/A |
| Kandiah et al, 2016 A | Number of CMBs:  Infratentorial | GRE | The Microbleed Anatomical Rating Scale^56^ | Mean=0.23,  SD=0.85 | Mean=0.15,  SD=0.68 | NS | 0.608 | Independent sample t-test or Wilcoxon-Mann-Whitney test | | No | N/A | N/A | N/A | N/A |
| Kandiah et al, 2016 A | Number of CMBs:  Total | GRE | The Microbleed Anatomical Rating Scale^56^ | Mean=1.69,  SD=4.43 | Mean=1.18,  SD=3.68 | NS | 0.145 | Independent sample t-test or Wilcoxon-Mann-Whitney test | | No* | N/A | N/A | N/A | N/A |
| Kandiah et al, 2016 B | Number of CMBs:  Cortical | GRE | The Microbleed Anatomical Rating Scale^56^ | Mean=0.49,  SD=1.25 | Mean=0.25,  SD=1.13 | NS | 0.097 | Independent sample t-test or Wilcoxon-Mann-Whitney test | | No* | N/A | N/A | N/A | N/A |
| Kandiah et al, 2016 B | Number of CMBs:  Sublobar | GRE | The Microbleed Anatomical Rating Scale^56^ | Mean=0.37  SD=0.97 | Mean=0.29  SD=1.11 | NS | 0.123 | Independent sample t-test or Wilcoxon-Mann-Whitney test | | No | N/A | N/A | N/A | N/A |
| Kandiah et al, 2016 B | Number of CMBs:  Infratentorial | GRE | The Microbleed Anatomical Rating Scale^56^ | Mean=0.23  SD=0.69 | Mean=0.13  SD=0.58 | NS | 0.324 | Independent sample t-test or Wilcoxon-Mann-Whitney test | | No | N/A | N/A | N/A | N/A |
| Makin et al, 2018 | Number of CMBs | NS | Defined according to STRIVE criteria^54^ | Median = 0,  IQR = 0-0 | Median = 0,  IQR = 0-0 | OR=1.02,  95% CI = 0.92-1.1 | 0.65 | Logistic regression | | No* | OR = 1.05,  95% CI = 0.93-1.16 | 0.29 | Logistic regression | No* |

# **Supplement 9: Lacunes**

**eTable 9.1: PRESENCE of lacunes and post-stroke DEMENTIA**

|  | | | | | | **Unadjusted analysis** | | | | **Adjusted analysis** | | | |
| --- | --- | --- | --- | --- | --- | --- | --- | --- | --- | --- | --- | --- | --- |
| **Study** | **Neuroimaging feature** | **MRI sequence** | **Measuring technique** | **Developed**  **PSD** | **Not developed PSD** | **Effect size** | **Sig. level** | **Statistical test** | **Associated with PSD** | **Effect size** | **Sig. level** | **Statistical test** | **Associated with PSD** |
| Moulin et al, 2016 | Presence of ≥1 old lacune | FLAIR | ‘Deep, subcortical, or pontine ovoid lesions (3-15mmm), with cerebrospinal fluid-like signal with or without a hyperintense FLAIR border’ | 50% | 34% | OR=1.96,  95% ci=1.02-3.75§ | 0.04 | Logistic regression  (Calculated by review authors) | Yes* | SHR=1.48, CI=0.86-2.54 | 0.16 | Fine-Gray model | No* |
| Xiong et al, 2019 | Presence of lacunes | FLAIR | ‘round or ovoid fluid-filled cavities between 3 to 15 mm in diameter’ | NS | NS | HR=0.676,  95% CI=0.252-1.816 | 0.437 | Univariable Cox proportional hazards analysis | No* | N/A | N/A | N/A | N/A |

*finding visualised in the harvest plot (Figure 2)

§data included in the meta-analysis

Abbreviations: N/A, not applicable, NS, not stated

**eTable 9.2: PRESENCE of lacunes and post-stroke COGNITIVE IMPAIRMENT**

|  | | | | | | **Unadjusted analysis** | | | | **Adjusted analysis** | | | |
| --- | --- | --- | --- | --- | --- | --- | --- | --- | --- | --- | --- | --- | --- |
| **Study** | **Neuroimaging feature** | **MRI sequence** | **Measuring technique** | **Developed**  **PSCI** | **Not developed PSCI** | **Effect size** | **Sig. level** | **Statistical test** | **Associated with PSCI** | **Effect size** | **Sig. level** | **Statistical test** | **Associated with PSCI** |
| Chen et al, 2016 | Presence of lacunes | FLAIR & T1 | “Small old infarcts, termed as lacunes, were CSF-like lesions of between 3mm and 15mm in diameter, with a surrounding rim of hyperintensity” | 81% | 72% | Cohen’s d=0.20 | 0.446 | Chi-square test | No* | N/A | N/A | N/A | N/A |
| Chen et al, 2016 | Presence of lacunes | FLAIR & T1 | “Small old infarcts, termed as lacunes, were CSF-like lesions of between 3mm and 15mm in diameter, with a surrounding rim of hyperintensity” | 81% | 72% | OR=1.62,  95%CI=0.47-5.64§ | 0.45 | Logistic regression  (Calculated by review authors) | No | N/A | N/A | N/A | N/A |
| Fruhwirth et al, 2020 | Pesence of ≥1 lacunes | NS | NS | NS | NS | N/A | N/A | N/A | N/A | Β=0.25 | 0.038 | Multiple linear regression | Yes* |
| Han et al, 2021 | Presence of lacunes | T1/T2/FLAIR | ‘There was a lacuna under the round or oval cortex, 3mm<*D*< 15 mm, and full  of fluid’ | NS | NS | OR presented in figure 9. Unable to extract specific OR. | <0.05 | Single factor binary logistic regression | Yes* | OR presented in figure 10. Unable to extract specific OR. | NS | Multivariate binary logistic regression analysis (adjusted for other neuroimaging features) | No* |
| Kandiah et al, 2016 A | Presence of ≥2 chronic lacunes | T1/T2 | ‘focal, discrete areas of apparent ischemic infarction … measuring 5-15mm at largest diameter.’ | 47% | 24% | NS | <0.001 | X^2^ test or Fisher’s Exact test | Yes* | β coefficient=0.859,  SE=0.398 | 0.031  (scoring 0,2) | Multivariate logistic regression | Yes* |
| Liang et al, 2019 | Presence of lacunae | NS | STRIVE criteria^54^ | NS | NS | N/A | N/A | N/A | N/A | OR = 1.45,  95% CI = 0.91-2.31 | 0.12 | Generalised estimating equation model  (Table 2 – model 4) | No* |
| Molad et al, 2019 | Presence of lacunes | T1 & T2 | ‘sharply  demarcated hypointense lesions sized between 3mm  and 15mm in diameter on ‘ | 41% | 37% | NS | 0.507 | Table 1  X^2^ test | No* | N/A | N/A | N/A | N/A |
| Molad et al, 2019 | Presence of lacunes | T1 & T2 | ‘sharply  demarcated hypointense lesions sized between 3mm  and 15mm in diameter’ | 41% | 37% | OR=1.18,  95% CI=0.72-1.95§ | 0.51 | Logistic regression  (Calculated by review authors) | No | N/A | N/A | N/A | N/A |

*finding visualised in the harvest plot (Figure 2)

§data included in the meta-analysis

Abbreviations: N/A, not applicable, NS, not stated

**eTable 9.3: NUMBER of lacunes and post-stroke COGNITIVE IMPAIRMENT**

|  | | | | | | **Unadjusted analysis** | | | | **Adjusted analysis** | | | |
| --- | --- | --- | --- | --- | --- | --- | --- | --- | --- | --- | --- | --- | --- |
| **Study** | **Neuroimaging feature** | **MRI sequence** | **Measuring technique** | **Developed**  **PSCI** | **Not developed PSCI** | **Effect size** | **Sig. level** | **Statistical test** | **Associated with PSCI** | **Effect size** | **Sig. level** | **Statistical test** | **Associated with PSCI** |
| Chen et al, 2016 | Number of lacunes | FLAIR & T1 | ‘between 3mm and 15mm in diameter, with a surrounding rim of hyperintensity’ | Mean:4.4, SD:4.2 | Mean:3.0, SD:3.3 | Cohen’s d=0.37 | 0.180 | Independent sample t-test | No* | NS | NS | Multivariate logistic regression | No* |
| Kandiah et al, 2016 A | Total number of chronic infarcts | T1/T2 | ‘focal, discrete areas of apparent ischemic infarction … measuring 5-15mm at largest diameter.’ | Mean=1.65,  SD=1.80 | Mean=0.99,  SD=1.71 | NS | <0.001 | Independent sample t-test or Wilcoxon-Mann-Whitney test | Yes* | N/A | N/A | N/A | N/A |
| Kandiah et al, 2016 A | Number of chronic infarcts: Cortical | T1/T2 | ‘focal, discrete areas of apparent ischemic infarction … measuring 5-15mm at largest diameter.’ | Mean=0.60,  SD=1.00 | Mean=0.29,  SD=0.72 | NS | 0.009 | Independent sample t-test or Wilcoxon-Mann-Whitney test | Yes | N/A | N/A | N/A | N/A |
| Kandiah et al, 2016 A | Number of chronic infarcts: Sublobar | T1/T2 | ‘focal, discrete areas of apparent ischemic infarction … measuring 5-15mm at largest diameter.’ | Mean=0.77,  SD=1.13 | Mean=0.49,  SD=0.92 | NS | 0.022 | Independent sample t-test or Wilcoxon-Mann-Whitney test | Yes | N/A | N/A | N/A | N/A |
| Kandiah et al, 2016 A | Number of chronic infarcts: Infratentorial | T1/T2 | ‘focal, discrete areas of apparent ischemic infarction … measuring 5-15mm at largest diameter.’ | Mean=0.28,  SD=0.66 | Mean=0.21,  SD=0.69 | NS | 0.187 | Independent sample t-test or Wilcoxon-Mann-Whitney test | No | N/A | N/A | N/A | N/A |
| Kandiah et al, 2016 B | Number of chronic infarcts: Cortical | T1/T2 | ‘focal, discrete areas of apparent ischemic infarction … measuring 5-15mm at largest diameter.’ | Mean=1.09,  SD=2.52 | Mean=0.44,  SD=0.85 | NS | 0.403 | Independent sample t-test or Wilcoxon-Mann-Whitney test | No* | N/A | N/A | N/A | N/A |
| Kandiah et al, 2016 B | Number of chronic infarcts: Sublobar | T1/T2 | .’ ‘focal, discrete areas of apparent ischemic infarction … measuring 5-15mm at largest diameter.’ | Mean=1.49,  SD=1.96 | Mean=0.77,  SD=1.13 | NS | 0.042 | Independent sample t-test or Wilcoxon-Mann-Whitney test | Yes | N/A | N/A | N/A | N/A |
| Kandiah et al, 2016 B | Number of chronic infarcts:Infratentorial | T1/T2 | ‘focal, discrete areas of apparent ischemic infarction … measuring 5-15mm at largest diameter.’ | Mean=0.34,  SD=0.64 | Mean=0.25,  SD=0.54 | NS | 0.411 | Independent sample t-test or Wilcoxon-Mann-Whitney test | No | N/A | N/A | N/A | N/A |

*finding visualised in the harvest plot (Figure 2)

§data included in the meta-analysis

Abbreviations: N/A, not applicable, NS, not stated

**eTable 9.4: LOCATIONS of lacunes and post-stroke COGNITIVE IMPAIRMENT**

|  | | | | | | **Unadjusted analysis** | | | | **Adjusted analysis** | | | |
| --- | --- | --- | --- | --- | --- | --- | --- | --- | --- | --- | --- | --- | --- |
| **Study** | **Neuroimaging feature** | **MRI sequence** | **Measuring technique** | **Developed**  **PSCI** | **Not developed PSCI** | **Effect size** | **Sig. level** | **Statistical test** | **Associated with PSCI** | **Effect size** | **Sig. level** | **Statistical test** | **Associated with PSCI** |
| Chen et al, 2016 | Location of lacunes: basal ganglia | FLAIR & T1 | ‘between 3mm and 15mm in diameter, with a surrounding rim of hyperintensity’ | 73% | 50% | Cohen’s d=0.40 | 0.145 | Chi-square test | No | N/A | N/A | N/A | N/A |
| Chen et al, 2016 | Location of lacunes: thalamus | FLAIR & T1 | ‘between 3mm and 15mm in diameter, with a surrounding rim of hyperintensity’ | 43% | 39% | Cohen’s d=0.19 | 0.488 | Chi-square test | No | N/A | N/A | N/A | N/A |
| Chen et al, 2016 | Location of lacunes: frontal lobe | FLAIR & T1 | ‘between 3mm and 15mm in diameter, with a surrounding rim of hyperintensity’ | 60% | 35% | Cohen’s d=0.30 | 0.269 | Chi-square test | No* | N/A | N/A | N/A | N/A |
| Chen et al, 2016 | Location of lacunes: temporal lobe | FLAIR & T1 | ‘between 3mm and 15mm in diameter, with a surrounding rim of hyperintensity’ | 3% | 12% | Cohen’s d=0.34 | 0.314 | Chi-square test | No | N/A | N/A | N/A | N/A |
| Chen et al, 2016 | Location of lacunes: parietal-occipital lobe | FLAIR & T1 | ‘between 3mm and 15mm in diameter, with a surrounding rim of hyperintensity’ | 17% | 4% | Cohen’s d=0.40 | 0.210 | Chi-square test | No | N/A | N/A | N/A | N/A |
| Chen et al, 2016 | Location of lacunes: infratentorial region | FLAIR & T1 | ‘between 3mm and 15mm in diameter, with a surrounding rim of hyperintensity’ | 13% | 8% | Cohen’s d=0.16 | 0.682 | Chi-square test | No | N/A | N/A | N/A | N/A |

*finding visualised in the harvest plot (Figure 2)

§data included in the meta-analysis

Abbreviations: N/A, not applicable, NS, not stated

**eTable 9.5: SIZE of lacunes and post-stroke COGNITIVE IMPAIRMENT**

|  | | | | | | **Unadjusted analysis** | | | | **Adjusted analysis** | | | |
| --- | --- | --- | --- | --- | --- | --- | --- | --- | --- | --- | --- | --- | --- |
| **Study** | **Neuroimaging feature** | **MRI sequence** | **Measuring technique** | **Developed**  **PSCI** | **Not developed PSCI** | **Effect size** | **Sig. level** | **Statistical test** | **Associated with PSCI** | **Effect size** | **Sig. level** | **Statistical test** | **Associated with PSCI** |
| Chen et al, 2016 | Lacunar size | FLAIR & T1 | ‘between 3mm and 15mm in diameter, with a surrounding rim of hyperintensity’ | Mean=7.6mm, SD=4.48mm | Mean=5.7mm, SD=4.3mm | Cohen’s d=0.44 | 0.107 | Independent sample t test | No* | NS | NS | Multivariate logistic regression | No* |

*finding visualised in the harvest plot (Figure 2)

§data included in the meta-analysis

Abbreviations: N/A, not applicable, NS, not stated

# **Supplement 10: Perivascular spaces**

**eTable 10.1: PRESENCE of perivascular spaces and post-stroke COGNITIVE IMPAIRMENT**

|  | | | | | | **Unadjusted analysis** | | | | **Adjusted analysis** | | | |
| --- | --- | --- | --- | --- | --- | --- | --- | --- | --- | --- | --- | --- | --- |
| **Study** | **Neuroimaging feature** | **MRI sequence** | **Measuring technique** | **Developed**  **PSCI** | **Not developed PSCI** | **Effect size** | **Sig. level** | **Statistical test** | **Associated with PSCI** | **Effect size** | **Sig. level** | **Statistical test** | **Associated with PSCI** |
| Molad et al, 2019 | Presence of perivascular spaces | T2 | ‘smooth margin, round, oval or linear shaped  lesions, sized up to 3 mm… enlarged  perivascular spaces at the level of the basal ganglia as  well as at the level of centrum semiovale in the most  involved hemisphere were counted’ | 89% | 93% | NS | 0.241 | Chi-square test | No* | N/A | N/A | N/A | N/A |

*finding visualised in the harvest plot (Figure 2)

§data included in the meta-analysis

Abbreviations: N/A, not applicable, NS, not stated

**eTable 10.2: NUMBER of perivascular spaces and post-stroke COGNITIVE IMPAIRMENT**

|  | | | | | | **Unadjusted analysis** | | | | **Adjusted analysis** | | | |
| --- | --- | --- | --- | --- | --- | --- | --- | --- | --- | --- | --- | --- | --- |
| **Study** | **Neuroimaging feature** | **MRI sequence** | **Measuring technique** | **Developed**  **PSCI** | **Not developed PSCI** | **Effect size** | **Sig. level** | **Statistical test** | **Associated with PSCI** | **Effect size** | **Sig. level** | **Statistical test** | **Associated with PSCI** |
| Han et al, 2021 | Number of PVS | T1/FLAIR/T2 | ‘The gap surrounded the blood vessel or run parallel to the blood vessel, in a line,  round, or oval, D< 3 mm’ | NS | NS | OR is visualised in a figure. | <0.05 | Single factor binary logistic regression | Yes* | OR is visualised in a figure. | <0.05 | Multivariate binary logistic regression analysis | Yes* |

*finding visualised in the harvest plot (Figure 2)

§data included in the meta-analysis

Abbreviations: N/A, not applicable, NS, not stated

**eTable 10.3: Perivascular space SCORE and post-stroke DEMENTIA**

|  | | | | | | **Unadjusted analysis** | | | | **Adjusted analysis** | | | |
| --- | --- | --- | --- | --- | --- | --- | --- | --- | --- | --- | --- | --- | --- |
| **Study** | **Neuroimaging feature** | **MRI sequence** | **Measuring technique** | **Developed**  **PSD** | **Not developed PSD** | **Effect size** | **Sig. level** | **Statistical test** | **Associated with PSD** | **Effect size** | **Sig. level** | **Statistical test** | **Associated with PSD** |
| **Xiong et al, 2019** | Centrum semiovale-enlarged perivascular space (score) | NS | 4-point visual rating scale^57^ | NS | NS | HR=0.862,  95% CI=0.524-1.418 | 0.558 | Univariable Cox proportional hazards analysis | No* | N/A | N/A | N/A | N/A |
| Liang et al, 2019 | Scores of enlarged perivascular spaces | NS | ‘One point was awarded to perivascular spaces when more  than 10 were located on one side of a single slice in the basal  ganglia’ | NS | NS | N/A | N/A | N/A | N/A | OR = 1.12,  CI = 0.95-1.32 | 0.17 | Generalised estimating equation model | No* |

*finding visualised in the harvest plot (Figure 2)

§data included in the meta-analysis

Abbreviations: N/A, not applicable, NS, not stated

# **Supplement 11: Cerebral small vessel disease**

**eTable 11.1: cSVD SCORE and post-stroke DEMENTIA**

|  | | | | | | **Unadjusted analysis** | | | | **Adjusted analysis** | | | |
| --- | --- | --- | --- | --- | --- | --- | --- | --- | --- | --- | --- | --- | --- |
| **Study** | **Neuroimaging feature** | **MRI sequence** | **Measuring technique** | **Developed**  **PSD** | **Not developed PSD** | **Effect size** | **Sig. level** | **Statistical test** | **Associated with PSD** | **Effect size** | **Sig. level** | **Statistical test** | **Associated with PSD** |
| **Xiong et al, 2019** | SVD score for cerebral amyloid angiopathy  (≥3 vs <3) | T1, T2, FLAIR | ‘2–4 CMBs, focal cSS, CSO-EPVS ≥20,  Fazekas ≥2  in deep WMH or Fazekas 3 in periventricular WMH  accounted for 1 point respectively, whereas >4 CMBs  and disseminated cSS accounted for 2 points.’ | NS | NS | HR=2.858,  95% CI=1.263-6.468 | 0.012 | Univariable Cox proportional hazards analysis | Yes*  (higher total SVD score for CAA) | HR=2.961,  95% CI=1.278-6.861 | 0.011 | Multivariable Cox proportional hazards regression | Yes* |

*finding visualised in the harvest plot (Figure 2)

§data included in the meta-analysis

Abbreviations: N/A, not applicable, NS, not stated

**eTable11.2: cSVD SCORE and post-stroke COGNITIVE IMPAIRMENT**

|  | | | | | | **Unadjusted analysis** | | | | **Adjusted analysis** | | | |
| --- | --- | --- | --- | --- | --- | --- | --- | --- | --- | --- | --- | --- | --- |
| **Study** | **Neuroimaging feature** | **MRI sequence** | **Measuring technique** | **Developed**  **PSCI** | **Not developed PSCI** | **Effect size** | **Sig. level** | **Statistical test** | **Associated with PSCI** | **Effect size** | **Sig. level** | **Statistical test** | **Associated with PSCI** |
| Coutureau et al, 2021 | SVD score | FLAIR/T1/T2*/SWI | ‘One point allocated for periventricular WMH Fazekas 3 or deep WMH Fazekas 2-3, presence of lacunes, moderate to severe (>10) perivascular spaces in basal ganglia, and presence of microbleeds’ | NS | N/S | OR=1.30,  95% CI=1.00-1.50§ | 0.05 | Univariate logistic regression | No* | OR=1.04,  95% CI=0.82-1.31§ | 0.7638 | Multivariate logistic regression | No* |
| Han et al, 2021 | CSVD score | T1/T2/FLAIR/  SWI/T2*GRE | Presence of each feature equates to 1 point  DWMH (Fazekas 2/3 points) and/or  PVWMH (Fazekas 3 points);  ≥1 CMB;  PVS in the basal ganglia of grades 2–4;  ≥1 lacuna | NS | NS | OR is visualised in a figure. | <0.05 | Single factor binary logistic regression | Yes* | OR is visualised in a figure. | <0.05 | Multivariate binary logistic regression analysis | Yes* |
| Liang et al, 2019 | SVD score | NS | Staals et al.^58^ | NS | NS | β=-0.33,  SE=0.13 | 0.008 | Univariate linear mixed model | Yes* | OR = 1.24,  95% CI = 1.06-1.44§ | 0.006 | Generalised estimating equation model | Yes* |
| Makin et al, 2018 | SVD score | NS | STRIVE criteria^54^ | Median = 2,  IQR = 1-2 | Median = 1,  IQR = 0-2 | OR = 1.46,  95% CI = 1.06-2.04§ | 0.02 | Logistic regression | Yes* | OR = 1.68,  95% CI = 1.05-2.76§ | 0.03 | Logistic regression | Yes* |
| Sung et al, 2021 | Modified CSVD burden | NS | Modified version of scale in Staals et al.^58^ | NS | NS | <3: Ref  ≥3:  B=-0.99,  SE=1.22 | 0.419 | Generalised estimating equation model | No* | N/A | N/A | N/A | N/A |
| Sung et al, 2021 | Status of modified CSVD score and hippocampal atrophy | NS | Modified version of scale in Staals et al.^58^ | NS | NS | N/A | N/A | N/A | N/A | mCSVD <3 and no HA: REF  mCSVD ≥3 and HA: B=-0.50, SE=1.28 (I think this should say and ‘no HA’)  mCSVD <3 and HA: B=-1.29, SE=1.42  mCSVD ≥3 and no HA: B=-2.85, SE=2.05 | mCSVD ≥3 and HA: 0.698  mCSVD <3 and HA: 0.365;  mCSVD ≥3 and no HA: 0.164 | Generalised estimating equation model | No |
| Zhi et al, 2021 | SVD score | SPGR, T1, T2, T2-FLAIR, GRE-T2 * | ‘One point was allocated for each of the following manifestation: lacune, severe periventricular (Fazekas score 3) or moderate-to severe deep WMH (Fazkeas score 2 or 3), deep microbleeds, or moderate-tosevere EPVS (>10) in basal ganglia.’ | 0: 16%;  1: 20%;  2: 22%;  3: 27%;  4: 15% | 0: 19%;  1: 29%;  2: 26%;  3:18%;  4; 8% | Overall cognition:  Non-standardised coefficients: -0.185,  95% CI=-0.303—0.068  Other cognitive test data are provided | 0.002 | Linear regression analysis | Yes*^[[36]](#footnote-36)^ | Overall cognition:  Non standardised coefficient: -0.190,  95% CI=-0.315 - -0.064 | 0.003 | Multivatiable regression analysis | Yes* |
| Zhi et al, 2021 | Total SVD score | SPGR, T1, T2, T2-FLAIR, GRE-T2* | Group 1: Total or modified SVD score = 0 point.;  Group 2:Total or modified SVD score = 1-2 point(s).;  Group 3:Total or modified SVD score = 3-4 points. | NS | NS | VCI:  Group 1: OR=1.00 (ref);  Group 2: OR=0.941, 95% CI=0.388-2.283;  Group 3: OR=2.000, 95%=0.769-5.198 | Ptrend=0.086 | Linear regression analysis  (Table 6) | No | N/A | N/A | N/A | N/A |

*finding visualised in the harvest plot (Figure 2)

§data included in the meta-analysis

Abbreviations: N/A, not applicable, NS, not stated

# **Supplement 12: Pre-existing stroke lesions**

**eTable 12.1: PRESENCE of pre-existing stroke lesions and post-stroke DEMENTIA**

|  | | | | | | **Unadjusted analysis** | | | | **Adjusted analysis** | | | |
| --- | --- | --- | --- | --- | --- | --- | --- | --- | --- | --- | --- | --- | --- |
| **Study** | **Neuroimaging feature** | **MRI sequence** | **Measuring technique** | **Developed**  **PSD** | **Not developed PSD** | **Effect size** | **Sig. level** | **Statistical test** | **Associated with PSD** | **Effect size** | **Sig. level** | **Statistical test** | **Associated with PSD** |
| Moulin et al, 2016 | Presence of ≥1 old macrohaemorrhage | T2*GRE weighted images | ‘>10mm in diameter distinct from the acute haemorrhage’ | 37%, | 17% | OR=2.83,  95% CI=1.38-5.82 | <0.01 | Logistic regression  (Calculated by review authors) | Yes* | SHR=2.9, CI=1.66-5.07 | 0.0002 | Fine-Gray model | Yes |

*finding visualised in the harvest plot (Figure 2)

§data included in the meta-analysis

Abbreviations: N/A, not applicable, NS, not stated

**eTable 12.2: PRESENCE of pre-existing stroke lesions and post-stroke COGNITIVE IMPAIRMENT**

|  | | | | | | **Unadjusted analysis** | | | | **Adjusted analysis** | | | |
| --- | --- | --- | --- | --- | --- | --- | --- | --- | --- | --- | --- | --- | --- |
| **Study** | **Neuroimaging feature** | **MRI sequence** | **Measuring technique** | **Developed**  **PSCI** | **Not developed PSCI** | **Effect size** | **Sig. level** | **Statistical test** | **Associated with PSCI** | **Effect size** | **Sig. level** | **Statistical test** | **Associated with PSCI** |
| Chen et al, 2016 | Presence of large old infarct | NS | NS | 19% | 4% | Cohen’s d=0.47 | 0.091 | Chi-square test | No | N/A | N/A | N/A | N/A |
| Mandzia et al, 2016 | Previous lacunar infarct | NS | STRIVE criteria^54^ | NS | NS | NS | Executive function:NS  Psychomotor Processing Speed: NS  Memory: NS | ‘Comparisons were made using t test/ANOVAs, Wilcoxon Tank-sum test, Fisher exact test, or Spearman correlation depending on type and distribution of variables’ | No | N/A | N/A | N/A | N/A |
| Mandzia et al, 2016 | Presence of previous cortical infarct | NS | STRIVE criteria^54^ | Impaired executive function: 20%;  Impaired psychomotor processing speed: 25%;  Impaired memory: 83%; | Normal executive function 4%;  Normal psychomotor processing speed: 7%;  Normal memory: 7%; | NS | Executive function:0.05  Psychomotor Processing Speed: 0.008  Memory: 0.99 | ‘Comparisons were made using t test/ANOVAs, Wilcoxon Tank-sum test, Fisher exact test, or Spearman correlation depending on type and distribution of variables’ | Presence of previous cortical infarct was associated with impaired psychomotor processing speed | Executive function: β -1,  SE:0.45,  t=-2.3,  95%CI -1.9,-0.1;  Psychomotor processing speed:  β-1.7,  SE:0.50,  t=-2.3,  95% CI -2,-0.2 | Executive function: 0.03;  Psychomotor processing speed: 0.02 | General linear modelling | Presence of previous cortical infarct was associated with impaired executive function and psychomotor processing speed |
| Fruhwirth et al, 2020 | Presence of ≥1 old cortical infarcts | NS | NS | NS | NS | NS | N/A | N/A | N/A | MoCA:  β=-0.04 | 0.764^[[37]](#footnote-37)^ | Multiple linear regression | No |
| Liang et al, 2019 | Old infarcts | NS | ‘NS | NS | NS | β -0.57,  SE=0.32 | 0.08 | Univariate linear mixed model | No | N/A | N/A | N/A | N/A |

*finding visualised in the harvest plot (Figure 2)

§data included in the meta-analysis

Abbreviations: N/A, not applicable, NS, not stated

**eTable 12.3: LOCATION of pre-existing stroke lesions and post-stroke COGNITIVE IMPAIRMENT**

|  | | | | | | **Unadjusted analysis** | | | | **Adjusted analysis** | | | |
| --- | --- | --- | --- | --- | --- | --- | --- | --- | --- | --- | --- | --- | --- |
| **Study** | **Neuroimaging feature** | **MRI sequence** | **Measuring technique** | **Developed**  **PSCI** | **Not developed PSCI** | **Effect size** | **Sig. level** | **Statistical test** | **Associated with PSCI** | **Effect size** | **Sig. level** | **Statistical test** | **Associated with PSCI** |
| Chen et al, 2016 | Location of large old infarcts: Basal ganglia | NS | NS | 7% | 4% | Cohen’s d=0.11 | 1.000 | Chi-square test | No | N/A | N/A | N/A | N/A |
| Chen et al, 2016 | Location of large old infarcts: frontal lobe | NS | NS | 3% | 0% | Cohen’s d=0.24 | 1.000 | Chi-square test | No | N/A | N/A | N/A | N/A |
| Chen et al, 2016 | Location of large old infarcts: parietal-occipital lobe | NS | NS | 7% | 0% | Cohen’s d=0.35 | 0.497 | Chi-square test | No | N/A | N/A | N/A | N/A |
| Chen et al, 2016 | Location of large old infarcts: infratentorial region | NS | NS | 7% | 0% | Cohen’s d=0.35 | 0.497 | Chi-square test | No | N/A | N/A | N/A | N/A |

*finding visualised in the harvest plot (Figure 2)

§data included in the meta-analysis

Abbreviations: N/A, not applicable, NS, not stated

# **Supplement 13: Acute stroke features**

**eTable 13.1: TYPE of stroke and post-stroke COGNITIVE IMPAIRMENT**

|  | | | | | | **Unadjusted analysis** | | | | **Adjusted analysis** | | | |
| --- | --- | --- | --- | --- | --- | --- | --- | --- | --- | --- | --- | --- | --- |
| **Study** | **Neuroimaging feature** | **MRI sequence** | **Measuring technique** | **Developed**  **PSCI** | **Not developed PSCI** | **Effect size** | **Sig. level** | **Statistical test** | **Associated with PSCI** | **Effect size** | **Sig. level** | **Statistical test** | **Associated with PSCI** |
| Chaudhari et al, 2014 | Ischaemic stroke | NS | IS vs ICH | IS: 87%; | IS:89% | NS | 0.716 | Chi-square | No* | N/A | N/A | N/A | N/A |
| Chaudhari et al, 2014 | Ischaemic stroke | NS | IS vs ICH | IS: 87%; | IS:89% | OR=0.80,  95%CI=0.24-2.67§ | 0.72 | Logistic regression  (Calculated by review authors) | No | N/A | N/A | N/A | N/A |
| Schellhorn et al, 2021 | Ischaemic stroke | NS | IS vs ICH | IS: 91% | IS: 95% | OR=0.49,  95% Ci=0.17-1.45§ | 0.2 | Logistic regression  (Calculated by review authors) | No* | N/A | N/A | N/A | N/A |

*finding visualised in the harvest plot (Figure 2)

§data included in the meta-analysis

Abbreviations: N/A, not applicable, NS, not stated

**eTable 13.2: PRESENCE/NUMBER/LOCATION of acute stroke lesion and post-stroke DEMENTIA**

|  | | | | | | **Unadjusted analysis** | | | | **Adjusted analysis** | | | |
| --- | --- | --- | --- | --- | --- | --- | --- | --- | --- | --- | --- | --- | --- |
| **Study** | **Neuroimaging feature** | **MRI sequence** | **Measuring technique** | **Developed**  **PSD** | **Not developed PSD** | **Effect size** | **Sig. level** | **Statistical test** | **Associated with PSD** | **Effect size** | **Sig. level** | **Statistical test** | **Associated with PSD** |
| Kumral et al, 2020 | Strategic lesions | T1/T2/FLAIR/DWI | “These strategic sites included large foci (.1.5 cm)  within the following regions: the middle and inferior frontal  gyri, parietal region, middle temporal gyrus, the putamen  and pallidum, cingulate gyrus, corpus callosum, arcuate  fasciculus, middle occipital region and hippocampus, thalamus,  midbrain, and postero-inferior cerebellum.” | Present=53%,  Absent=48% | Present=15%,  Absent=85% | OR=6.42, 95%CI=5.59-7.38 | 0.0001 | Logistic regression | Yes | OR=3.27, 95%CI=2.76-3.88 | 0.0001 | Multiple logistic regression | Yes |
| Kumral et al, 2020 | Stroke lesion volume | NS | STRIVE criteria^54^ | NS | NS | OR=1.04,  95% CI=1.034-1.038 | 0.0001 | Logistic regression | Yes | OR=1.044, 95%CI=1.035-1.054 | 0.0001 | Multiple logistic regression | Yes |
| Kumral et al, 2020 | Single infarct present | DWI/FLAIR | Single infarct | 22% | 70% | NS | 0.0001 | Chi-square | Yes | N/A | N/A | N/A | N/A |
| Kumral et al, 2020 | Multiple infarcts present | DWI/FLAIR | ‘at least two diffusion-weighted imaging or FLAIR lesions in at least two different arterial territories' | 78% | 30% | NS | 0.0001 | Chi-square | Yes | N/A | N/A | N/A | N/A |
| Kumral et al, 2020 | Multiple acute infarcts in one cerebral hemisphere | DWI/FLAIR | ‘infarcts… involving the territories  of the middle cerebral artery, the anterior cerebral artery,  and the anterior choroidal artery’ | 40% | 14% | OR=4.16, 95%CI=3.62-4.78 | 0.0001 | Logistic regression | Yes | OR=2.59, 95%CI=1.40-4.80 | 0.002 | Multiple logistic regression | Yes |
| Kumral et al, 2020 | Multiple acute bihemispheric infarcts in the anterior circulation | DWI/FLAIR | ‘Multiple acute bihemispheric  infarcts in the anterior circulation were presumed in  patients with acute multiple infarcts involving both hemispheres  with lesions either in superficial or deep arterial  territories.’ | 14% | 6% | OR=2.37, 95%CI=1.94-2.90 | 0.0001 | Logistic regression | Yes | OR=2.33, 95%CI=1.31-4.15 | 0.001 | Multiple logistic regression | Yes |
| Kumral et al, 2020 | Multiple acute infarcts in the posterior circulation | DWI/FLAIR | ‘Multiple acute infarcts in the posterior circulation  were determined in patients with concomitant infarcts  in the territory of the vertebral arteries, the basilar artery,  the superior cerebellar artery, the anterior inferior cerebellar  artery, the posterior inferior cerebellar artery, and the  posterior cerebral artery.’ | 14% | 7% | OR=2.26, 95%CI=1.86-2.75 | 0.0001 | Logistic regression | Yes | OR=2.41, 95%CI=1.89-3.07 | NS | Multiple logistic regression | NS |
| Kumral et al, 2020 | Multiple acute infarcts in the anterior and posterior circulation, | DWI/FLAIR | ‘Multiple acute infarcts in the anterior  and posterior circulation were considered in patients  with combined supratentorial and infratentorial infarcts.’ | 10% | 4% | OR=3.17, 95%CI=2.51-3.99 | 0.0001 | Logistic regression | Yes | N/A | N/A | N/A | N/A |
| Lin et al, 2003 | Infarct location | T1 | Cortical;  Subcortical;  Unknown | Cortical: 42%;  Subcortical: 58%;  Unknown: 0% | Cortical: 19%;  Subcortical: 77%;  Unknown: 4% | X^2^=7.90 | 0.02 | Chi-square test | Yes  (Cortical lesions were associated) | NS | NS | Stepwise logistic regression | No  (Cortical lesions were not associated with PSD) |
| Lin et al, 2003 | Vascular territory | T1 | Left carotid;  Right carotid;  Vertebrobasilar;  Unknown | Left carotid: 77%;  Right carotid: 19%;  Vertebrobasilar: 4%;  Unknown: 0% | Left carotid: 36%;  Right carotid: 26%;  Vertebrobasilar: 33%;  Unknown: 4% | X^2^=18.0 | 0.001 | Chi-square test | Yes  (Left carotid lesions were associate with PSD) | Left carotid:  β=2.53,  SE=1.11,  OR=12.5,  95% CI=1.4-110.2;  Right carotid:  β=1.61,  SE=1.119,  OR=5.0, 95% CI=0.5-51.5;  Vertebrobasilar & unknown:  OR=1.0 (reference) | Left carotid: 0.0226;  Right carotid: 0.1776  Vertebrobasilar & unknown: reference | Stepwise logistic regression | Yes  (Left carotid vascular territory was associated with PSD) |
| **Xiong et al, 2019** | Intraventricular haemorrhage involvement (yes vs no) | NS | NS | NS | NS | HR=1.062,  95% CI=0.398-2.833 | 0.905 | Univariable Cox proportional hazards analysis | No | N/A | N/A | N/A | N/A |

*finding visualised in the harvest plot (Figure 2)

§data included in the meta-analysis

Abbreviations: N/A, not applicable, NS, not stated

**eTable 13.3: PRESENCE/NUMBER/LOCATION of acute stroke lesion and post-stroke COGNITIVE IMPAIRMENT**

|  | | | | | | **Unadjusted analysis** | | | | **Adjusted analysis** | | | |
| --- | --- | --- | --- | --- | --- | --- | --- | --- | --- | --- | --- | --- | --- |
| **Study** | **Neuroimaging feature** | **MRI sequence** | **Measuring technique** | **Developed**  **PSCI** | **Not developed PSCI** | **Effect size** | **Sig. level** | **Statistical test** | **Associated with PSCI** | **Effect size** | **Sig. level** | **Statistical test** | **Associated with PSCI** |
| **Chaudhari et al, 2014** | Dominant hemispheric stroke | NS | ‘involvement of dominant vs. non-dominant hemisphere’ | 44% | 38% | NS | 0.540 | Chi-square | No | N/A | N/A | N/A | N/A |
| **Chaudhari et al, 2014** | Strategic site lesion | T2-FLAIR/ DWI/ ADC | ‘strategic site of the  lesion that was defined as the lesions involving areas like thalamus,  angular gyrus, caudate, globus pallidus, basal forebrain, cingulate  gyrus, genu or anterior limb of internal capsule or hippocampus’ | 44% | 11% | NS | <0.001 | Chi-square | Yes  (Strategic site lesion associated with PSCI) | OR: 22.857,  95% CI: 2.911-179.498 | 0.003 | Binary logistic regression | Yes  (Strategic site lesion was associated with PSCI) |
| Schellhorn et al, 2021 | Left hemispheric stroke | NS | NS | 51% | 38% | OR=1.80,  95% CI=1.05-3.09 | 0.032 | Univariate logistic regression | Yes | OR=2.33,  95% CI=1.26-4.34 | 0.007 | Multiple logistic regression | Yes |
| Schiemanck et al, 2005 | Mixed or subcortical lesions | T2 and/or FLAIR | “Lesions affecting cortical grey matter only and lesions affecting cortical grey matter with subcortical structures involved were classified as “mixed” lesions. If cortex was not affected, and only subcortical structures were involved, lesions were defined as purely “subcortical” lesions.” | NS | NS | NS | NS | NS | No | NS | NS | Spearman rank correlation | Yes |
| Sivakumar et al, 2017 | Presence of lacunar infarcts | FLAIR | STRIVE criteria^54^ | 32% | Transient deficits=24%;  No deficits=18% | NS | 0.091 | Logistic regression  (study compared three cognitive groups) | No | N/A | N/A | N/A | N/A |
| Sivakumar et al, 2017 | Presence of lacunar infarcts | FLAIR | STRIVE criteria^54^ | 32% | Transient deficits=24%;  No deficits=18% | OR=2.02,  95% CI=0.67-6.12 | 0.21 | Logistic regression  (Calculated by review authors) | No | N/A | N/A | N/A | N/A |

*finding visualised in the harvest plot (Figure 2)

§data included in the meta-analysis

Abbreviations: N/A, not applicable, NS, not stated

**eTable 13.4: NUMBER of lesions and post-stroke DEMENTIA**

|  | | | | | | **Unadjusted analysis** | | | | **Adjusted analysis** | | | |
| --- | --- | --- | --- | --- | --- | --- | --- | --- | --- | --- | --- | --- | --- |
| **Study** | **Neuroimaging feature** | **MRI sequence** | **Measuring technique** | **Developed**  **PSD** | **Not developed PSD** | **Effect size** | **Sig. level** | **Statistical test** | **Associated with PSD** | **Effect size** | **Sig. level** | **Statistical test** | **Associated with PSD** |
| Lin et al, 2003 | Number of lesions | T1 | Multiple;  Single | Multiple: 85%;  Single: 15% | Multiple: 65%;  Single: 35% | X^2^=3.96 | 0.05 | Chi-square test | No | N/A | N/A | N/A | N/A |

*finding visualised in the harvest plot (Figure 2)

§data included in the meta-analysis

Abbreviations: N/A, not applicable, NS, not stated

# **Supplement 14: Additional neuroimaging features**

**eTable 14.1: Additional neuroimaging features and post-stroke DEMENTIA**

|  | | | | | | **Unadjusted analysis** | | | | **Adjusted analysis** | | | |
| --- | --- | --- | --- | --- | --- | --- | --- | --- | --- | --- | --- | --- | --- |
| **Study** | **Neuroimaging feature** | **MRI sequence** | **Measuring technique** | **Developed**  **PSD** | **Not developed PSD** | **Effect size** | **Sig. level** | **Statistical test** | **Associated with PSD** | **Effect size** | **Sig. level** | **Statistical test** | **Associated with PSD** |
| **Xiong et al, 2019** | Presence of cortical superficial siderosis | GRE | ‘cSS status was categorized into absence, focal (restricted to ≤3 sulci), or disseminated (affecting 4 or more sulci)’ | NS | NS | HR=2.300,  95% CI=1.045-5.064 | 0.039 | Univariable Cox proportional hazards analysis | Yes | N/A | N/A | N/A | N/A |
| **Xiong et al, 2019** | Focal cSS versus no cSS | GRE | ‘cSS status was categorized into absence, focal (restricted to ≤3 sulci), or disseminated (affecting 4 or more sulci)’ | NS | NS | HR=1.763,  95% CI=0.675-4.609 | 0.247 | Univariable Cox proportional hazards analysis | No | N/A | N/A | N/A | N/A |
| **Xiong et al, 2019** | Presence of disseminated cSS | GRE | ‘cSS status was categorized into absence, focal (restricted to ≤3 sulci), or disseminated (affecting 4 or more sulci)’ | NS | NS | HR=3.590,  95% CI=1.271-10.135 | 0.016 | Univariable Cox proportional hazards analysis | Yes | HR=3.275,  95% CI=1.129-9.499 | 0.029 | Multivariable Cox proportional hazards regression | Yes |
| Moulin et al, 2016 | Presence of superficial siderosis | T2*-GRE/FLAIR | ‘Cortical superficial siderosis was defined as a homogeneous  curvilinear signal intensity (black) on T2*-GRE  sequences in the superficial layers of the cerebral cortex,  within the subarachnoid space, away from at least two  sulci of the haematoma’ | Any superficial siderosis = 33%;  Focal superficial siderosis = 12%;  Disseminated superficial siderosis = 23% | Any superficial siderosis = 13%;  Focal superficial siderosis = 12%;  Disseminated superficial siderosis = 2% | NS | NS | NS | NS | Any superficial siderosis = SHR=2.31, CI=1.27-4.20;  Focal superficial siderosis = ShR=0.98, CI=0.44-2.17;  Disseminated superficial siderosis = SHR=4.10, CI=1.91-8.79 | Any superficial siderosis = 0.006;  Focal superficial siderosis = 0.96;  Disseminated superficial siderosis = 0.0003 | Fine-Gray model | Yes  (any superficial siderosis and disseminated superficial siferosis) |
| Moulin et al, 2016 | Disseminated superficial siderosis | T2*-GRE/FLAIR | ‘Cortical superficial siderosis was defined as a homogeneous  curvilinear signal intensity (black) on T2*-GRE  sequences in the superficial layers of the cerebral cortex,  within the subarachnoid space, away from at least two  sulci of the haematoma’ | Disseminated superficial siderosis = 23% | Disseminated superficial siderosis = 2% | NS | NS | NS | NS | SHR=7.45, CI=4.27-12.99 | 0.0001 | Backward stepwise multivariable analysis | Yes |

*finding visualised in the harvest plot (Figure 2)

§data included in the meta-analysis

Abbreviations: N/A, not applicable, NS, not stated

**eTable 14.2: Additional neuroimaging features and post-stroke COGNITIVE IMPAIRMENT**

|  | | | | | | **Unadjusted analysis** | | | | **Adjusted analysis** | | | |
| --- | --- | --- | --- | --- | --- | --- | --- | --- | --- | --- | --- | --- | --- |
| **Study** | **Neuroimaging feature** | **MRI sequence** | **Measuring technique** | **Developed**  **PSCI** | **Not developed PSCI** | **Effect size** | **Sig. level** | **Statistical test** | **Associated with PSCI** | **Effect size** | **Sig. level** | **Statistical test** | **Associated with PSCI** |
| Banergee et al, 2019 | Evidence of haemorrhagic transformation | T2*GRE | ECASS classification | NS | NS | NS | NS | Chi square/Fisher’s exact | No | NS | NS | NS | NS |
| Molad et al, 2019 | Presence of lacunes and/or microbleeds | Lacunes: T1 & T2;  Microbleeds: SWI/T1/DWI | Lacunes: ‘sharply  demarcated hypointense lesions sized between 3mm  and 15mm in diameter’;  Microbleeds: ‘round hypointense lesions  on SWI with a diameter <10 mm’ | NS | NS | HR=1.58,  95% CI=0.96-5.6 | 0.069 | Logistic regression | No | HR=1.5,  95%CI=1.82-2.72 | 0.194 | Multivariate Cox regression | No |

*finding visualised in the harvest plot (Figure 2)

§data included in the meta-analysis

Abbreviations: N/A, not applicable, NS, not stated

# **Supplement 15: Sensitivity Analysis**

| **Unadjusted** | | | | | | | | | | | | | | | | |
| --- | --- | --- | --- | --- | --- | --- | --- | --- | --- | --- | --- | --- | --- | --- | --- | --- |
|  | **Patients without pre-stroke dementia and/or cognitive impairment only** | | | | **Patients with ischaemic stroke only** | | | | **Cognitive outcome diagnosed at least six months after the index stroke only** | | | | **Cognition assessed using neuropsychological battery or diagnostic criteria** | | | |
| **MRI feature** | **No. of studies** | **No. of patients** | **OR**  **(95% CI)** | **Sig. of pooled OR** | **No. of studies** | **No. of patients** | **OR**  **(95% CI)** | **Sig. of pooled OR** | **No. of studies** | **No. of patients** | **OR**  **(95% CI)** | **Sig. of pooled OR** | **No. of studies** | **No. of patients** | **OR**  **(95% CI)** | **Sig. of pooled OR** |
| Presence of cerebral atrophy | 3^4, 17, 26A^ | 453 | 2.48,  (1.33-4.62) | **0.004** | 2^4, 26A^ | 265 | 2.12  (1.27-3.55) | **0.004** | 2^4, 17^ | 244 | 3.28  (1.23-8.77) | **0.02** | **2^4, 17^** | 244 | 3.28  (1.23-8.77) | **0.02** |
| cSVD | 2^6, 14^ | 499 | 1.34  (1.12-1.61) | **0.001** | 2^6, 14^ | 499 | 1.34  (1.12-1.61) | **0.001** | 1^14^ | Insufficient number of studies | | | 0 | Insufficient number of studies | | |
| Presence of WMH | 3^11, 17, 20^ | 8993 | 2.35  (0.92-6.01) | 0.07 | 1^11^ | Insufficient number of studies | | | 1^17^ | Insufficient number of studies | | | 2^11, 17^ | 8888 | 2.08  (0.55-7.88) | 0.28 |
| Presence of lacunes | 2^4, 17^ | 244 | 1.88  (1.06-3.35) | **0.03** | 1^4^ | Insufficient number of studies | | | 3^4, 16, 17^ | 641 | 1.46  (0.96-2.23) | 0.08 | 3^4, 16, 17^ | 641 | 1.46  (0.96-2.23) | 0.08 |
| Isch stroke vs ICH | 1^3^ | Insufficient number of studies | | | N/A | | | | N/A | | | | 2^3, 18^ | 333 | 0.61  (0.27-1.39) | 0.24 |

Includes studies reporting PSD and PSCI

| **Adjusted** | | | | | | | | | | | | | | | | |
| --- | --- | --- | --- | --- | --- | --- | --- | --- | --- | --- | --- | --- | --- | --- | --- | --- |
|  | **Patients without pre-stroke dementia and/or cognitive impairment only** | | | | **Patients with ischaemic stroke only** | | | | **Cognitive outcome diagnosed at least six months after the index stroke only** | | | | **Cognition assessed using neuropsychological battery or diagnostic criteria** | | | |
| **MRI feature** | **No. of studies** | **No. of patients** | **OR**  **(95% CI)** | **Sig. of pooled OR** | **No. of studies** | **No. of patients** | **OR**  **(95% CI)** | **Sig. of pooled OR** | **No. of studies** | **No. of patients** | **OR**  **(95% CI)** | **Sig. of pooled OR** | **No. of studies** | **No. of patients** | **OR**  **(95% CI)** | **Sig. of pooled OR** |
| cSVD score | 3^6, 12, 14^ | 950 | 1.23  (0.96-1.57) | 0.11 | 3^6, 12, 14^ | 950 | 1.23  (0.96-1.57) | 0.11 | 2^12, 14^ | 602 | 1.33  (1.02-1.75) | **0.04** | 0 | Insufficient number of studies | | |
| Presence of CMB | 2^11, 12^ | 9151 | 1.36  (1.08-1.70) | **0.009** | 2^11, 12^ | 9151 | 1.36  (1.08-1.70) | **0.009** | 1^12^ | Insufficient number of studies | | | 1^11^ | Insufficient number of studies | | |
| WMH score | 3^3, 12, 14^ | 704 | 1.26  (1.06-1.49) | **0.008** | 2^12, 14^ | 602 | 1.26  (0.96-1.66) | 0.11 | 2^12, 14^ | 602 | 1.26  (0.96-1.66) | 0.11 | 1^3^ | Insufficient number of studies | | |

Includes studies reporting PSD and PSCI

# **Supplement 16: Meta-analysis of risk factors associated with PSCI/PSD (unadjusted prognostic factors)**

| **Risk factors** | **No. of studies** | **No. of patients** | **OR (95% CI)** | **Sig. of pooled**  **OR** | **I squared** | **p-value for heterogeneity** |
| --- | --- | --- | --- | --- | --- | --- |
| **MRI Neuroimaging features** | | | | | | |
| Presence of cerebral atrophy | 3^4, 17, 26A^ | 453 | **2.48 (1.33-4.62)** | **0.004** | 0% | 0.49 |
| Presence of WMH | 3^11, 17, 20^ | 8993 | 2.35 (0.92-6.01 | 0.07 | 72% | 0.03 |
| Presence of lacunes | 3^4, 16, 17^ | 641 | 1.46 (0.96-2.23) | 0.08 | 0% | 0.47 |
| cSVD score | 2^6, 14^ | 499 | **1.34 (1.12-1.61)** | **0.001** | 0% | 0.55 |
| Ischemic stroke (vs haemorrhagic stroke) | 2^3, 18^ | 333 | 0.61 (0.27-1.39) | 0.24 | 0% | 0.55 |
| **Demographic factors** | | | | | | |
| Female | 12^3, 4, 11, 13, 14, 16, 18, 20, 24, 25, 26A, 26B^ | 10,679 | 1.25 (0.83-1.90) | 0.29 | 51% | 0.02 |
| Low education | 4^3, 13, 25, 26A^ | 697 | **3.80 (1.89-7.62)** | **<0.001** | 66% | 0.03 |
| **Vascular factors** | | | | | | |
| Hypertension | 12^3, 4, 11, 13, 14, 16, 18, 20, 24, 25, 26A, 26B^ | 10,679 | 1.49 (0.94-2.37) | 0.09 | 70% | <.001 |
| Diabetes | 12^3, 4, 11, 13, 14, 16, 18, 20, 24, 25, 26A, 26B^ | 10,679 | 1.21 (0.87-1.70) | 0.26 | 64% | 0.001 |
| Atrial fibrillation | 6^4, 11, 14, 25, 26A, 26B^ | 9404 | **2.41 (1.24-4.72)** | **0.010** | 54% | 0.05 |
| Hypercholesterolemia | 10^3, 4, 11, 13, 16, 18, 20, 24, 26A, 26B^ | 10,425 | **1.41 (1.03-1.95)** | **0.03** | 45% | 0.06 |
| Smoking | 10^3, 4, 11, 14, 16, 18, 24, 25, 26A, 26B^ | 10,291 | 0.79 (0.62-1.01) | 0.06 | 0% | 0.44 |
| High alcohol intake | 4^3, 4, 14, 24^ | 315 | 0.58 (0.30-1.12) | 0.11 | 0% | 0.42 |
| Ischemic heart disease | 6^3, 4, 13, 14, 26A, 26B^ | 986 | 0.99 (0.52-1.90) | 0.98 | 7% | 0.37 |
| Prior stroke | 7^3, 4, 13, 18, 25, 26A, 26B^ | 1169 | **2.62 (1.49-4.60)** | **<.001** | 58% | 0.03 |
| Prior TIA | 3^11, 26A, 26B^ | 9094 | 1.19 (0.37-3.82) | 0.77 | 78% | 0.01 |
| APOE | 2^11, 16^ | 9097 | **1.47 (1.09-1.97)** | **0.01** | 9% | 0.29 |

*Bold values indicate statistically significant result

References

1. Appelros P, Samuelsson M, Lindell D. Lacunar infarcts: functional and cognitive outcomes at five years in relation to MRI findings. Cerebrovascular diseases. 2005;20(1):34-40.

2. Banerjee G, Chan E, Ambler G, et al. Effect of small-vessel disease on cognitive trajectory after atrial fibrillation-related ischaemic stroke or TIA. Journal of Neurology. 2019 May;266(5):1250-9.

3. Chaudhari TS, Verma R, Garg RK, et al. Clinico-radiological predictors of vascular cognitive impairment (VCI) in patients with stroke: a prospective observational study. Journal of the neurological sciences. 2014 May 15;340(1-2):150-8.

4. Chen X, Duan L, Han Y, et al. Predictors for vascular cognitive impairment in stroke patients. BMC neurology. 2016 Dec;16(1):1-8.

5. Christ N, Mocke V, Fluri F. Cerebral microbleeds are associated with cognitive decline early after ischemic stroke. Journal of Neurology. 2019 May;266(5):1091-4.

6. Coutureau J, Asselineau J, Perez P, et al. Cerebral small vessel disease MRI features do not improve the prediction of stroke outcome. Neurology. 2021 Jan 26;96(4):e527-37.

7. Fruhwirth V, Enzinger C, Fandler‐Höfler S, et al. Baseline white matter hyperintensities affect the course of cognitive function after small vessel disease‐related stroke: a prospective observational study. European Journal of Neurology. 2021 Feb;28(2):401-10.

8. Gregoire SM, Smith K, Jäger HR, et al. Cerebral microbleeds and long-term cognitive outcome: longitudinal cohort study of stroke clinic patients. Cerebrovascular diseases. 2012;33(5):430-5.

9. Han L, Liu L, Hao Y, et al. Diagnosis and treatment effect of convolutional neural network-based magnetic resonance image features on severe stroke and mental state. Contrast Media & Molecular Imaging. 2021 Jul 26;2021.

10. Kang HJ, Stewart R, Park MS, et al. White matter hyperintensities and functional outcomes at 2 weeks and 1 year after stroke. Cerebrovascular diseases. 2013;35(2):138-45.

11. Kumral E, Bayam FE, Arslan H, et al. Associations Between Neuroanatomic Patterns of Cerebral Infarctions and Vascular Dementia. The Journal of Neuropsychiatry and Clinical Neurosciences. 2021 Jan;33(1):49-56.

12. Liang Y, Chen YK, Liu YL, et al. Cerebral small vessel disease burden is associated with accelerated poststroke cognitive decline: a 1-year follow-up study. Journal of Geriatric Psychiatry and Neurology. 2019 Nov;32(6):336-43.

13. Lin JH, Lin RT, Tai CT, et al. Prediction of poststroke dementia. Neurology. 2003 Aug 12;61(3):343-8.

14. Makin SD, Doubal FN, Shuler K, et al. The impact of early-life intelligence quotient on post stroke cognitive impairment. European stroke journal. 2018 Jun;3(2):145-56.

15. Mandzia JL, Smith EE, Horton M, et al. Imaging and baseline predictors of cognitive performance in minor ischemic stroke and patients with transient ischemic attack at 90 days. Stroke. 2016 Mar;47(3):726-31.

16. Molad J, Hallevi H, Korczyn AD, et al. Vascular and neurodegenerative markers for the prediction of post-stroke cognitive impairment: results from the TABASCO study. Journal of Alzheimer's Disease. 2019 Jan 1;70(3):889-98.

17. Moulin S, Labreuche J, Bombois S, et al. Dementia risk after spontaneous intracerebral haemorrhage: a prospective cohort study. The Lancet Neurology. 2016 Jul 1;15(8):820-9.

18. Schellhorn T, Aamodt EB, Lydersen S, et al. Clinically accessible neuroimaging predictors of post-stroke neurocognitive disorder: a prospective observational study. BMC neurology. 2021 Dec;21(1):1-1.

19. Schiemanck SK, Post MW, Kwakkel G, et al. Ischemic lesion volume correlates with long-term functional outcome and quality of life of middle cerebral artery stroke survivors. Restorative neurology and neuroscience. 2005 Jan 1;23(3-4):257-63.

20. Sivakumar L, Riaz P, Kate M, et al. White matter hyperintensity volume predicts persistent cognitive impairment in transient ischemic attack and minor stroke. International Journal of Stroke. 2017 Apr;12(3):264-72.

21. Sung PS, Lee KP, Lin PY, et al. Factors Associated with Cognitive Outcomes After First-Ever Ischemic Stroke: The Impact of Small Vessel Disease Burden and Neurodegeneration. Journal of Alzheimer's Disease. 2021 Jan 1;83(2):569-79.

22. Xiong L, Charidimou A, Pasi M, et al. Predictors for late post-intracerebral hemorrhage dementia in patients with probable cerebral amyloid angiopathy. Journal of Alzheimer's Disease. 2019 Jan 1;71(2):435-42.

23. Zhang Q, Wang A, Meng X, et al. Vascular Risk Factors, Imaging, and Outcomes in Transient Ischemic Attack/Ischemic Stroke Patients with Neuroimaging Evidence of Probable/Possible Cerebral Amyloid Angiopathy. Oxidative Medicine and Cellular Longevity. 2021 Apr 26;2021.

24. Zhi N, Zhang L, Wang Y, et al. Modified cerebral small vessel disease score is associated with vascular cognitive impairment after lacunar stroke. Aging (Albany NY). 2021 Apr 15;13(7):9510.

25. Zhong HH, Qu JF, Xiao WM, et al. Severity of Lesions Involving the Cortical Cholinergic Pathways May Be Associated With Cognitive Impairment in Subacute Ischemic Stroke. Frontiers in neurology. 2021 Jun 8;12:606897.

26. Kandiah N, Chander RJ, Lin X, et al. Cognitive impairment after mild stroke: Development and validation of the SIGNAL 2 risk score. Journal of Alzheimer's Disease. 2016 Jan 1;49(4):1169-77.

27. Auriel E, Kliper E, Shenhar-Tsarfaty S, et al. Impaired renal function is associated with brain atrophy and poststroke cognitive decline. Neurology. 2016 May 24;86(21):1996-2005.

28. Ben Assayag E, Eldor R, Korczyn AD, et al. Type 2 diabetes mellitus and impaired renal function are associated with brain alterations and poststroke cognitive decline. Stroke. 2017 Sep;48(9):2368-74.

29. Chander RJ, Lim L, Handa S, et al. Atrial fibrillation is independently associated with cognitive impairment after ischemic stroke. Journal of Alzheimer's Disease. 2017 Jan 1;60(3):867-75.

30. Kandiah N, Wiryasaputra L, Narasimhalu K, et al. Frontal subcortical ischemia is crucial for post stroke cognitive impairment. Journal of the neurological sciences. 2011 Oct 15;309(1-2):92-5.

31. Liang Y, Chen YK, Liu YL, et al. Exploring causal pathways linking cerebral small vessel diseases burden to poststroke depressive symptoms with structural equation model analysis. Journal of Affective Disorders. 2019 Jun 15;253:218-23.

32. Molad J, Hallevi H, Korczyn AD, et al. The Interrelation Between Chronic Headache, Cognitive Scores, and MRI Markers Among Stroke Survivors. Journal of Alzheimer's Disease. 2021 Jan 1;81(4):1555-66.

33. Molad J, Kliper E, Korczyn AD, et al. Only white matter hyperintensities predicts post-stroke cognitive performances among cerebral small vessel disease markers: results from the TABASCO study. Journal of Alzheimer's Disease. 2017 Jan 1;56(4):1293-9.

34. Sagnier S, Catheline G, Munsch F, et al. Severity of Small Vessel Disease Biomarkers Reduces the Magnitude of Cognitive Recovery after Ischemic Stroke. Cerebrovascular Diseases. 2021;50(4):456-63.

35. Sagnier S, Okubo G, Catheline G, et al. Chronic cortical cerebral microinfarcts slow down cognitive recovery after acute ischemic stroke. Stroke. 2019 Jun;50(6):1430-6.

36. Schouten EA, Schiemanck SK, Brand N, et al. Long-term deficits in episodic memory after ischemic stroke: evaluation and prediction of verbal and visual memory performance based on lesion characteristics. Journal of stroke and cerebrovascular diseases. 2009 Mar 1;18(2):128-38.

37. Tang WK, Chen YK, Lu JY, Wong A, Mok V, Chu WC, Ungvari GS, Wong KS. Absence of cerebral microbleeds predicts reversion of vascular ‘cognitive impairment no dementia’in stroke. International Journal of Stroke. 2011 Dec;6(6):498-505.

38. Wang Z, van Veluw SJ, Wong A, et al. Risk factors and cognitive relevance of cortical cerebral microinfarcts in patients with ischemic stroke or transient ischemic attack. Stroke. 2016 Oct;47(10):2450-5.

39. Xiong YY, Wong A, Mok VC, et al. Frequency and predictors of proxy‐confirmed post‐stroke cognitive complaints in lacunar stroke patients without major depression. International journal of geriatric psychiatry. 2011 Nov;26(11):1144-51.

40. Yatawara C, Guevarra A, Ng KP, et al. Interactions between acute infarcts and cerebrovascular pathology predict poststroke dementia. Alzheimer Disease & Associated Disorders. 2020 Jul 29;34(3):206-11.

41. Yatawara C, Ng KP, Chander R, et al. Associations between lesions and domain-specific cognitive decline in poststroke dementia. Neurology. 2018 Jul 3;91(1):e45-54.

42. Kumral E, Güllüoğlu H, Alakbarova N, et al. Cognitive decline in patients with leukoaraiosis within 5 years after initial stroke. Journal of Stroke and Cerebrovascular Diseases. 2015 Oct 1;24(10):2338-47.

43. Narasimhalu K, Wiryasaputra L, Sitoh YY, et al. Post‐stroke subjective cognitive impairment is associated with acute lacunar infarcts in the basal ganglia. European Journal of Neurology. 2013 Mar;20(3):547-51.

44. Wang Z, Wong A, Liu W, et al. Cerebral microbleeds and cognitive function in ischemic stroke or transient ischemic attack patients. Dementia and Geriatric Cognitive Disorders. 2015;40(3-4):130-6.

45. Benedictus MR, Hochart A, Rossi C, et al. Prognostic factors for cognitive decline after intracerebral hemorrhage. Stroke. 2015 Oct;46(10):2773-8.

46. Mok V, Chang C, Wong A, et al. Neuroimaging determinants of cognitive performances in stroke associated with small vessel disease. Journal of neuroimaging. 2005 Apr;15(2):129-37.

47. McKhann, G.M., Knopman, D.S., Chertkow, H., et al, 2011. The diagnosis of dementia due to Alzheimer’s disease: Recommendations from the National Institute on Aging-Alzheimer’s Association workgroups on diagnostic guidelines for Alzheimer's disease. *Alzheimer's & dementia*, *7*(3), pp.263-269.

48. Ingles JL, Wentzel C, Fisk JD, et al. Neuropsychological predictors of incident dementia in patients with vascular cognitive impairment, without dementia. Stroke. 2002 Aug 1;33(8):1999-2002.

49. Pasquier F, Leys D, Weerts JG, et al. Inter-and intraobserver reproducibility of cerebral atrophy assessment on MRI scans with hemispheric infarcts. European neurology. 1996;36(5):268-72.

50. Scheltens P, Leys D, Barkhof F, et al. Atrophy of medial temporal lobes on MRI in" probable" Alzheimer's disease and normal ageing: diagnostic value and neuropsychological correlates. Journal of Neurology, Neurosurgery & Psychiatry. 1992 Oct 1;55(10):967-72.

51. Fazekas F, Niederkorn K, Schmidt R, et al. White matter signal abnormalities in normal individuals: correlation with carotid ultrasonography, cerebral blood flow measurements, and cerebrovascular risk factors. Stroke. 1988 Oct;19(10):1285-8.

52. Pantoni L, Basile AM, Pracucci G, et al. Impact of age-related cerebral white matter changes on the transition to disability–the LADIS study: rationale, design and methodology. Neuroepidemiology. 2005;24(1-2):51-62.

53. Wahlund LO, Barkhof F, Fazekas F, et al. A new rating scale for age-related white matter changes applicable to MRI and CT. Stroke. 2001 Jun;32(6):1318-22.

54. Wardlaw JM, Smith EE, Biessels GJ, et al. Neuroimaging standards for research into small vessel disease and its contribution to ageing and neurodegeneration. The Lancet Neurology. 2013 Aug 1;12(8):822-38.

55. Cordonnier C, Potter GM, Jackson CA, et al. Improving interrater agreement about brain microbleeds: development of the Brain Observer MicroBleed Scale (BOMBS). Stroke. 2009 Jan 1;40(1):94-9.

56. Gregoire SM, Chaudhary UJ, Brown MM, et al. The Microbleed Anatomical Rating Scale (MARS): reliability of a tool to map brain microbleeds. Neurology. 2009 Nov 24;73(21):1759-66.

57. Charidimou A, Jaunmuktane Z, Baron JC, et al. White matter perivascular spaces: an MRI marker in pathology-proven cerebral amyloid angiopathy?. Neurology. 2014 Jan 7;82(1):57-62..

58. Staals J, Makin SD, Doubal FN, et al. Stroke subtype, vascular risk factors, and total MRI brain small-vessel disease burden. Neurology. 2014 Sep 30;83(14):1228-34.

[**Reference 26:** 26A refers to the development cohort within this study. 26B refers to the validation cohort within this study.]

1. These studies stated that they recruited participants from settings including: hospitals, emergency department, particular hospital departments. [↑](#footnote-ref-1)
2. Lacunar stroke syndrome with lacunar infarct. [↑](#footnote-ref-2)
3. This study may have also used gadolinium. [↑](#footnote-ref-3)
4. Included people with ischemic cardioembolic stroke & TIA with non-valvular atrial fibrillation, eligible to start anticoagulation. [↑](#footnote-ref-4)
5. Excluded pre-stroke cognitive impairment [↑](#footnote-ref-5)
6. Excluded prior haemorrhagic stroke [↑](#footnote-ref-6)
7. This study reported two datasets. Data from Dataset 1 only has been extracted in this systematic review. Patients in Dataset 2 may have been included in a separate study which has been included in this review (Moulin et al, 2016). [↑](#footnote-ref-7)
8. Supratentorial cerebral infarct (NIHSS 1-25) [↑](#footnote-ref-8)
9. Excluded severe cognitive impairment (dementia) [↑](#footnote-ref-9)
10. Small subcortical infarction [↑](#footnote-ref-10)
11. Study states they excluded ‘patients with non-cerebrovascular conditions known to influence cognitive function’ [↑](#footnote-ref-11)
12. Severe stroke (NIHSS≥21) [↑](#footnote-ref-12)
13. Study states they excluded ‘patients with a family history of dementia but does not state excludes those with dementia’ [↑](#footnote-ref-13)
14. mRS≤2 [↑](#footnote-ref-14)
15. NIHSS<7 [↑](#footnote-ref-15)
16. No patients included in follow-up had a diagnosis of pre-stroke dementia or mild cognitive impairment. [↑](#footnote-ref-16)
17. IS (NIHSS<4) & TIA (high risk) [↑](#footnote-ref-17)
18. Mild to moderate ischaemic stroke [↑](#footnote-ref-18)
19. Parenchymal haemorrhage [↑](#footnote-ref-19)
20. Excluded pre-stroke dementia [↑](#footnote-ref-20)
21. Participants must have MCA lesion [↑](#footnote-ref-21)
22. NIHSS ≤ 3 [↑](#footnote-ref-22)
23. Excluded premorbid cognitive dysfunction and dementia [↑](#footnote-ref-23)
24. Lobar ICH with probable CAA [↑](#footnote-ref-24)
25. This is unclear. We understand N=291 were included in follow-up. [↑](#footnote-ref-25)
26. Lacunar stroke only [↑](#footnote-ref-26)
27. NIHSS<15 [↑](#footnote-ref-27)
28. N=4 had questionable dementia. [↑](#footnote-ref-28)
29. This study also compared VaD to VCIND. For the purpose of this review, we have extracted data comparing ‘VCI (includes VaD)’ to ‘No VCI’. [↑](#footnote-ref-29)
30. The study followed-up patients at 3-6 months and 12-18 months post-stroke. Study reported MRI features associated with cognitive assessment performed at 3-6 months. [↑](#footnote-ref-30)
31. For the purpose of this review, the review authors have considered PSCI to include patients with mild and major neurocognitive disorder. [↑](#footnote-ref-31)
32. For the purpose of this review, the review authors have considered PSCI to include patients with ‘persisting deficits’. Patients with ‘transient deficits’ and ‘no deficits’ were categorised as ‘No PSCI’ [↑](#footnote-ref-32)
33. MMSE used as a marker of overall cognition for some neuroimaging features. [↑](#footnote-ref-33)
34. Table 2 in the study outlines the association between presence/absence of CMBs and other cognitive domains. [↑](#footnote-ref-34)
35. This study reported deep and lobar CMB. We reported lobar CMB in the harvest plot as lobar CMB is more representative of total CMB. [↑](#footnote-ref-35)
36. Data relating to modified SVD score are also presented in the individual study paper [↑](#footnote-ref-36)
37. Table 4 in the study provides data for other cognitive domains. [↑](#footnote-ref-37)
